# What Evidence Is There for the Homology of Protein-Protein Interactions?

**DOI:** 10.1371/journal.pcbi.1002645

**Published:** 2012-09-20

**Authors:** Anna C. F. Lewis, Nick S. Jones, Mason A. Porter, Charlotte M. Deane

**Affiliations:** 1 Department of Statistics, University of Oxford, Oxford, United Kingdom; 2 Systems Biology Doctoral Training Centre, University of Oxford, Oxford, United Kingdom; 3 Department of Mathematics, Imperial College, London, United Kingdom; 4 Department of Physics, University of Oxford, Oxford, United Kingdom; 5 CABDyN Complexity Centre, University of Oxford, Oxford, United Kingdom; 6 Oxford Centre for Integrative Systems Biology, University of Oxford, Oxford, United Kingdom; 7 Oxford Centre for Industrial and Applied Mathematics, Mathematical Institute, University of Oxford, Oxford, United Kingdom; Princeton University, United States of America

## Abstract

The notion that sequence homology implies functional similarity underlies much of computational biology. In the case of protein-protein interactions, an interaction can be inferred between two proteins on the basis that sequence-similar proteins have been observed to interact. The use of transferred interactions is common, but the legitimacy of such inferred interactions is not clear. Here we investigate transferred interactions and whether data incompleteness explains the lack of evidence found for them. Using definitions of homology associated with functional annotation transfer, we estimate that conservation rates of interactions are low even after taking interactome incompleteness into account. For example, at a blastp 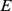-value threshold of 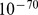, we estimate the conservation rate to be about 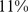 between *S. cerevisiae* and *H. sapiens*. Our method also produces estimates of interactome sizes (which are similar to those previously proposed). Using our estimates of interaction conservation we estimate the rate at which protein-protein interactions are lost across species. To our knowledge, this is the first such study based on large-scale data. Previous work has suggested that interactions transferred within species are more reliable than interactions transferred across species. By controlling for factors that are specific to within-species interaction prediction, we propose that the transfer of interactions within species might be less reliable than transfers between species. Protein-protein interactions appear to be very rarely conserved unless very high sequence similarity is observed. Consequently, inferred interactions should be used with care.

## Author Summary

It is widely assumed that knowledge gained in one species can be transferred to another species, even among species that are widely separated on the tree of life. This transfer is often done at the level of proteins under the assumption that if two proteins have similar sequences, they will share similar properties. In this paper, we investigate the validity of this assumption for the case of protein-protein interactions. The transfer of protein interactions across species is a common procedure and it is known to have shortcomings but these are generally ascribed to the incompleteness of protein interaction data. We introduce a framework to take such incomplete information into account, and under its assumptions show that the procedure is unreliable when using sequence-similarity thresholds typically thought to allow the transfer of functional information. Our results imply that, unless using strict definitions of homology, interactions rewire at a rate too fast to allow reliable transfer across species. We urge caution in interpreting the results of such transfers.

## Introduction

*Homology* – similarity through common descent – occurs on scales ranging, from genetic sequence to anatomy. The high degree of observed protein sequence homology gives a strong expectation that discoveries about protein function made in one species will provide understanding in another [1]. The extent of homology of protein function is of both practical and theoretical importance, as it underlies the reliance on a few model organisms and provides insight into the maintenance and diversification of protein function through evolution.

In this paper, we examine the evidence for homology in the realm of protein-protein interactions. Proteins, the main workhorses of the cell, do not carry out their functions in isolation but rather interact with each other to bring about biological function. In this study, we ask the following question: To what extent are protein-protein interactions conserved through evolution? A high degree of conservation makes viable the transfer of interactions across species. This is particularly pertinent given the cost of gathering experimental data and the concentration of that data in very few species. If, however, there is a low degree of conservation of protein interactions then – given the very high degree of conservation of protein sequences – this would suggest that interaction information cannot be transfered across species and that interactions can be lost and gained rapidly with little sequence change. This, in turn, could help explain how small changes in protein sequence on occasion bring about large phenotypic changes.

**Figure 1:**
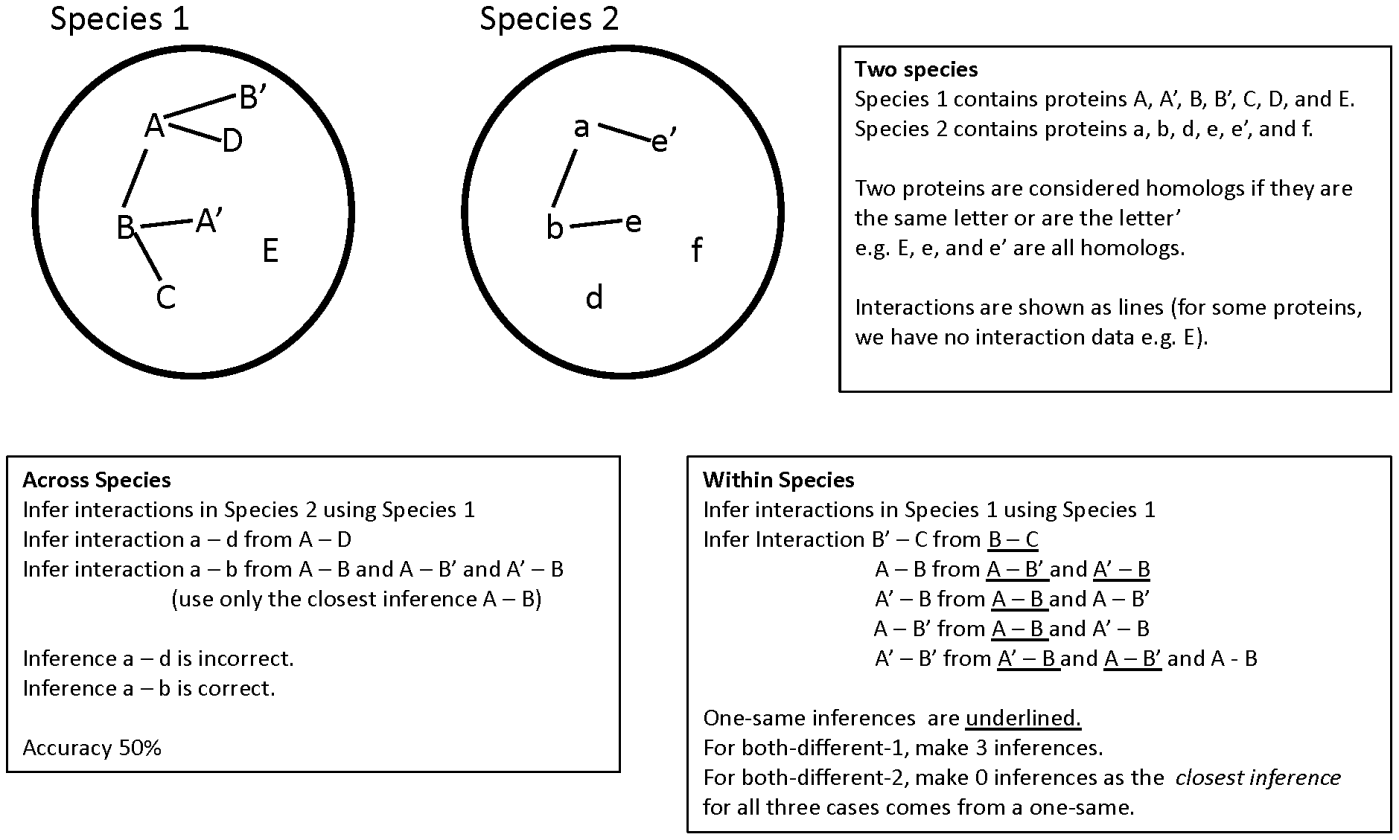
Methodology for infering protein-protein interactions.

The homology of protein-protein interactions can be investigated by seeking evidence of *interologs*. Interologs are pairs of interacting proteins: 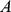 interacting with 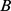 in one species and 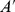 interacting with 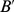 in another, where 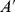 is a homolog of 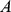 and 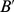 is a homolog of 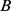 (see Figure 1). Homolog detection is an unsolved problem [2], so we consider three different definitions of homology: blastp [3] reciprocal hits at different thresholds of similarity, blastp reciprocal best hits, and EnsemblCompara GeneTrees [4].

The notion of across-species interologs was first introduced by Walhout et al in 2000 [5]. Since then, many studies have predicted interactions on the basis of transfer by homology (e.g. [6]–[18]). Despite the prevalent use of transferred interactions, relatively little work has been published that investigates the reliability of this procedure across species. Published success rates for transferring interactions vary from less than 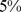 [19] to 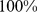 [20], and many values in between have been reported [8], [21]–[24]. These differences can be explained in part by methodological choices. For example, Qian et al [20] reported the highest conservation rate. They excluded gene-duplicates and compared two organisms that are evolutionarily very close. In contrast, the majority of studies have focused on comparisons between species that are much more distant on the tree of life – budding yeast *S. cerevisiae* (SC), nematode worm *C. elegans* (CE), fruitfly *D. melanogaster* (DM), and human *H. sapiens* (HS) – as these are the species for which there exists the most data [19], [21]–[23].

It is also possible to investigate the homology of interactions *within* a species. Two types of homologous interactions exist. Interactions 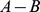 and 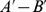 are homologous; we refer to these as *both-different* conserved interactions. Additionally, interactions 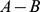 and 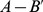 are homologous; we refer to such interactions as *one-same* conserved interactions. Mika and Rost found that interactions were more conserved within species than across species [23]. They considered this result surprising due to the long-standing belief that proteins arising from gene-duplication events (paralogs) must diverge in function in order to be conserved, whereas proteins that arise from a speciation event (orthologs) have evolutionary pressure to maintain the function of the ancestral protein [25]. However, Mika and Rost did not separate orthologs from paralogs in their across-species study so the results that they observed might be due to across-species out-paralogs outnumbering orthologs.

Errors in the interaction data, both – false negatives (i.e. existing interactions that are not reported in the data set) and false positives (i.e. interactions in the data set that do not actually exist) – can clearly have a substantial impact on results. Most obviously, false negatives in the target interactome will cause some interactions to be judged as non-conserved when the data in the target species is simply missing. However, except for Ref. [24], which examines one type of protein (transcription factors) in one pair of species (mouse and human), none of these studies investigated the role of errors in the data when assessing conservation.

A brief survey of the literature gives a sense of how significant these errors are believed to be. False-positive rates in high-throughput protein-protein interaction data, which have been estimated to be in excess of 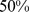 [26]–[28], have more recently been estimated at 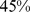 or considerably lower [29], [30]. False-positive rates in the multiple studies that are collated to give literature-curated data sets seem hard to assess. Error rates in the curation process have been estimated to be as high as 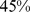 [31]. By comparing the estimated sizes of interactomes to the current sizes of data sets, false-negative rates of aggregate data sets can be derived. Recent estimates of the *S. cerevisiae* interactome range from 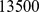 [32] to 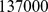 [29] interactions (c.f. 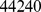 interactions in the data set we use); recent estimates for *H. sapiens* range from 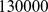 [30] to about 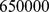 [32] (c.f. 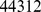 in our data set); and recent estimates for *D. melanogaster* range from about 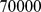 [32] to 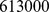 [29] (c.f. 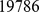 in our data set). *C. elegans* has been estimated to have about 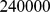 interactions [32] (c.f. 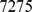 in our data set). The large range of estimates gives a flavour of how results depend on the assumptions made. These estimates indicate that the false-negative rates for all species except *S. cerevisiae* are very high, whereas the *S. cerevisiae* interactome is potentially nearly complete.

In addition to being far from 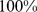 in all organisms save *S. cerevisiae*, the coverage of interactomes is biased [33], [34]. In particular, there is a high correlation between the number of publications in which a protein is mentioned and the number of interactions reported for that protein in literature-curated data (an 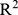 value of 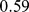 was reported by [34]). This reflects the fact that low-throughput experiments are hypothesis-driven, i.e. particular interactions are tested for if they are of interest to researchers. If hypotheses are formulated in part on what is known about homologous proteins, then one should expect a bias in which homologous interactions are more likely to be reported. This would lead to conservation rates appearing inflated compared to data sampled independently in different species.

In this study, we investigate the evidence for the homology of binary protein-protein interactions using data from six species: *S. cerevisiae* (SC), *C. elegans* (CE), *D. melanogaster* (DM), *H. sapiens* (HS), fission yeast *S. pombe* (SP) and mouse *M. musculus* (MM). The first four species we investigate because there exists considerable data for them, the last two because these species are evolutionarily close to *S. cerevisiae* and *H. sapiens* respectively, and thus represent an interesting point of comparison.

In the first part of the present study, we calculate observed conservation rates for interactions across species and discuss the effects of potential bias.

In the second part, we attempt to address the sources of error that could cause the observed conversation rates to be underestimates. We decouple the effects of interaction completeness from the conservation of interactions through evolution and thereby arrive at estimates for both. Using the assumptions of our model and definitions of homology frequently employed for transferring functional annotations, we show that the fraction of interactions that are conserved is low even when interactome errors are taken into account. If strict definitions of homology are employed, the number of conserved interactions across species is low. We emphasise that our estimates of the fraction of conserved interactions do not consider the biases in the interaction data and are hence probably *over*estimates. We then produce estimates for the rate at which interactions are lost through evolution – the first, to our knowledge, based on large-scale data sets and comparing species that are well separated on the tree of life – finding rates of about 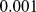 per million years between the most sequence-similar proteins.

In the third part of this study, we consider the transfer of interactions within-species. We examine three different sets of inferences. Set one is *one-same* inferences, where 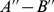 is inferred from 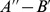 where 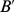 and 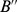 are homologs and 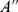 is present in both interactions. Set two is *both-different-1* inferences, for example, 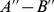 is inferred from 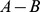 where 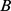 and 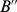 are homologs and 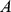 and 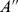 are homologs. In a final case study on this data (*both-different-2*) we identify the closest homologous interaction, and keep just a single inference for each interaction. This means if the closest inference comes from a one-same inference we no longer make a prediction from a less similar both-different inference. It has been shown previously that inferences of the one-same type are very powerful in within-species interaction prediction [23], a result we also observe. If one wishes to compare the rate of conservation of interactions within species to that across species then excluding one-same interactions as done in Ref. [23] seems fair. In our test of this type (both-different-1) we find that within-species interactions are conserved to approximately the same extent as across species interactions.

Functional annotations are often transferred using definitions that are not particularly strict (see, e.g., [35]–[37]). We argue that the low success of interaction transfer at comparable levels of sequence similarity cannot be explained solely by interactome errors. Unless a very stringent definition of homolog is employed, the rate of evolutionary change of interactions is too high to allow transfer across species that are well separated on the tree of life. At such stringent definitions, the number of conserved interactions is low. The common practice of transferring interactions on the basis of homology between such distant species [6]–[17] must be treated with caution.

## Results/Discussion

### Protein-protein interaction data

There are two primary types of protein-protein interactions: (1) direct protein-protein interaction data, which is reported predominantly via the yeast-two-hybrid screen and by small-scale studies and (2) evidence that proteins participate in the same complex, which is reported predominantly by Tandem Affinity Purification followed by Mass Spectroscopy experiments. (For a review of experimental techniques see Ref. [38].) These different types of interaction have a different nature; for example, they are predisposed to be identified between different protein functional classes [33]. As the ratios of direct protein-protein interactions to within-complex interactions differ substantially by species (within-complex data is concentrated within *S. cerevisiae* [39]–[42]), we investigate only direct protein-protein interactions. We amalgamate the interaction data from several sources (see Materials and Methods for details). Table 1 gives the data set sizes for the species that we investigate. This data combines results from low-throughput and high-throughput studies. We give an indication of the relative contributions of low- and high-throughput studies by calculating the fraction of interactions that are reported by a study that observed fewer than one hundred interactions. These relative contributions are not altered greatly if a different threshold is used (see Table S1 in Text S1); they reflect that large yeast-two-hybrid screens have been performed for *S. cerevisiae*, *C. elegans*, *D. melanogaster*, and *H. sapiens*, and that there has been a predictably large volume of small-scale experiments curated for *H. sapiens*. As indicated in Table 1, there are many more interactions per protein reported for *S. cerevisiae* than for any other species, and the interaction data for *S. pombe* and *M. musculus* are particularly sparse. Comparing the sizes of the interactomes of these data sets to the estimates of the total sizes of the interactomes surveyed in the Introduction, it is clear that the *S. cerevisiae* interactome might not be far from complete, whereas the coverage of the other interactomes is low.

**Table 1:** We assembled direct protein-protein interactions from BioGRID, IntAct, MINT, and HPRD (see Materials and Methods for details).

	SC	CE	DM	HS	MM	SP
# interactions	44266	7275	20334	45695	2911	1155
Fraction of low-throughput interactions	0.15	0.15	0.07	0.61	0.82	0.97
# proteins in interactome	5782	3988	6514	9597	2101	793
Mean # of interactions for proteins in interactome	7.6558	1.8242	3.1216	4.7614	1.3855	1.4565
# proteins (approximate)	6490	19522	13520	20763	21427	4806
Mean # of interactions for all proteins	6.8206	0.3727	1.504	2.2008	0.1359	0.2403

Low-throughput interactions are those interactions that have supporting evidence in publications that report fewer than one hundred interactions. (The trends are not sensitive to this choice of cut-off, see Table S1 in Text S1.) The *S. cerevisiae* network is more complete than those of the other species: a much higher fraction of *S. cerevisiae* proteins have protein-protein interaction data, and each protein is involved in more interactions. The approximate number of proteins only considers one protein isoform per gene. (We report the number of unique STRING identifiers; see Materials and Methods.)

### Homology data

Detecting homologs is an unsolved problem [2], so one must adopt some operational definition. Sequence similarity lies at the heart of judging whether sequences are homologous [43], though more advanced techniques incorporate additional information such as phylogenetic-tree analysis and gene-tree/species-tree reconciliation [2], [4], [44]. A conservative operational definition has the advantage that false-positive homologs will be minimised, but the disadvantage that many true homologs will be missed. In the context of inferring functional annotations from a source species to a target species, a conservative definition of homology will lead to low numbers of predictions. We consider three different operational definitions of homology: blastp [3] reciprocal hits; blastp reciprocal best hits; and EnsemblCompara GeneTrees [4]. Of these, reciprocal best hits is the most conservative and reciprocal hits is the least conservative.

The most common tool used to identify potentially homologous protein sequences on large scales is blastp [3]. Use of this method enables one to connect the success of interolog prediction with the blast 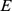-value, which is the most common diagnostic used to measure sequence similarity. The 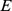-value (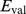) gives a measure of how often one would expect to observe a particular hit by chance when a query sequence is compared to a database of potential hit sequences. Reciprocal hits (see Materials and Methods) gives many-to-many homology relationships (i.e. each query sequence can have many hits). Rather than choosing a particular sequence-similarity cut-off, we investigate the success of interolog inferences at different 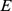-value thresholds. The least strict definition of homology we use is a blast 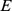-value of 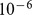. In the Supporting Information, we also give results for using different minimum percentage-sequence-identity of the aligned region values as an operational definition of homology. See Table S2 in Text S1 for the numbers of homologs found at two different 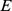-value thresholds: 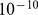 and the more stringent 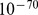.

The much more conservative set of reciprocal best hits gives one-to-one homology relationships (see Materials and Methods). We report the numbers of reciprocal-best-hit homologs in Table S3 in Text S1. EnsemblCompara GeneTrees [4] uses a gene-tree/species-tree reconciliation approach. We report the numbers of orthologs defined by EnsemblCompara GeneTrees in Table S4 in Text S1. EnsemblCompara GeneTrees does not include orthology relationships between *S. pombe* and non-fungi species. We also use the manually-curated orthologs between *S. pombe* and *S. cerevisiae* that are reported in Ref. [45]. There are 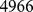 homology relationships reported between 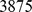 *S. cerevisiae* proteins and 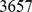 *S. pombe* proteins.

### Interactions conserved across species: the evidence

From an interaction 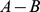 in the source species, we infer all interactions 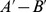 in the target species, where 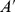 is a sequence homolog of 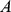 and 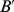 is a sequence homolog of 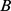 (see Figure 1). We consider all six species as source species but exclude *M. musculus* and *S. pombe* as target species because of the sparsity of data in these organisms. (We do, however, consider them as target species for *H. sapiens* and *S. cerevisiae*, respectively.) For the reciprocal-hit data, we investigate the effect of the 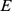-value as an operational definition of homology (meaning that both 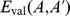 and 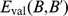 must be below a similarity threshold). Each interaction in the target species can conceivably be predicted more than once, but we consider only one inference to it. Hence, when we report the number of transferred interactions that are correct, we always give the number of unique interactions that are predicted correctly.

**Figure 2:**
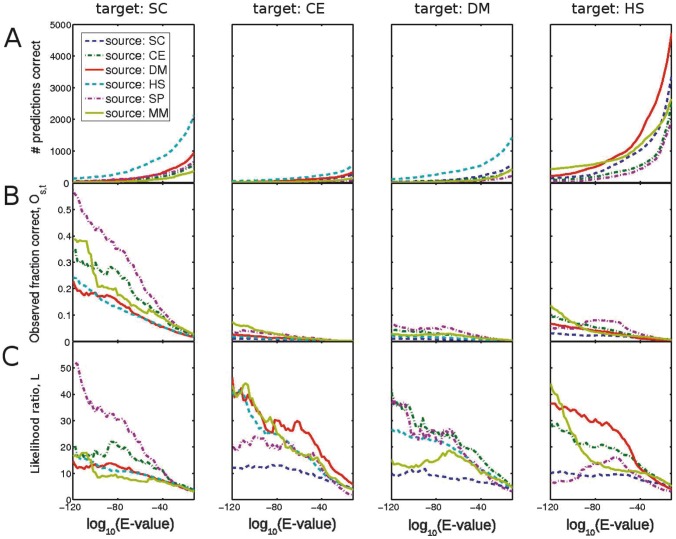
Large numbers of correct inferences are only observed when the fraction of correct inferences is very low. We show the results of inferring interactions from *S. cerevisiae* (SC), *C. elegans* (CE), *D. melanogaster* (DM), *H. sapiens* (HS), *S, Pombe* (SP), and *M. musculus* (MM) to the first four of those species. (A) Number of correct interolog inferences across species, for different blastp 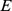-value cut-offs. (B) Fraction of all inferences that are observed in the interactions of the target species, 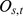. (C) The likelihood ratio 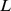 that an inference is correct. This indicates how much better it is to use the inferences than to select random pairs of proteins in the target species that have homologs in the source species interactome. (A) and (B) together indicate that it is only at lax 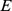-values that one makes significant numbers of correct inferences, but this is a very small fraction of the total number of inferences made at these 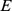-values. The *S. cerevisiae* data-set coverage is significantly higher than that of other species, so one obtains larger values for inferences to *S. cerevisiae*.

We compute the number of inferred interactions that are correct by counting how many of them are found in the interaction set of the target species (see Figure 2 A). The fraction of correct inferences observed, denoted 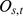, is the number of correct inferences divided by the total number of inferences (see Figure 2 B). As seen in Figure 2 A for the reciprocal-hits data, large numbers of correct inferences are made only at relatively lax 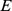-values (to the right side of the figure). However, as would be expected and shown in Figure 2 B, only a small fraction of the inferences are correct at these lax 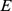-value cut-offs. (Figure S1 in Text S1 contains the same figure with the axes scaled differently for each target species. Figure S2 in Text S1 shows the same data but for thresholds of percentage sequence identity rather than for 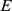-value.)

It is important to compare the success of inferring interactions using homology relative to that achieved with random guesses – i.e. how often randomly chosen pairs of proteins will actually interact. One must define what class of inferences are ‘random’: we first consider a random inference as one between any two proteins in the target species, given that they both have homologs in the source-species interactome. Figure 2 C gives the likelihood ratio 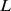 for an interolog to be a true prediction (see Materials and Methods). The likelihood that a transferred interaction is correct is only a few times better than random at lax 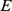-values, and it is not much larger even at very strict 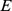-values (note very few correct predictions are made at such strict 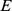-values). The likelihood is generally higher for inferences across species that diverged more recently. For example, inferences from *S. pombe* to *S. cerevisiae* have a higher likelihood than those between *S. cerevisiae* and other species. An alternative comparison to random inference is possible by rewiring the interactions in the source species while fixing the number of interactions for each protein (see Materials and Methods). This comparison controls for biases in protein appearance in the source-species interaction list. (Such biases could either result from the data-gathering process or reflect the underlying biology.) We give the ratio of the number of correct inferences from the actual source-species interactions to the mean of several random sets of interactions for each species pair in Table 2. A comparison between Figure 2 C and Table 2 illustrates that the different propensities for proteins to appear in the source species accounts for some of the success of transferring interactions on the basis of homology.

**Table 2:** Across species: How does inferring interactions from the source-species interactome compare to inferring interactions from randomised versions of the source-species interactome?

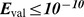					
target species		SC	CE	DM	HS
source species	SC	-	2.3 (0.17)	2.1 (0.091)	1.9 (0.092)
	CE	2.3 (0.18)	-	2.2 (0.16)	1.6 (0.13)
	DM	2.3 (0.10)	2.1 (0.076)	-	1.9 (0.047)
	HS	2.4 (0.068)	2.1 (0.047)	2.0 (0.072)	-
	MM	2.3 (0.25)	1.8 (0.18)	1.7 (0.44)	2.0 (0.37)
	SP	2.5 (0.21)	1.7 (0.22)	1.7 (0.19)	1.5 (0.092)

We give the ratio of the fraction of correct inferences 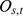 from the real interaction data compared to randomly rewired data for the reciprocal-hits homologs. (The number of interactions in which each protein participates is preserved in the randomization.) The numbers in parentheses give the standard deviations over 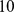 rewirings.

Although there are no standard 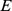-value thresholds that are used to define homology, we draw attention to two thresholds that often appear in the literature. A threshold of 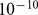 is considered a fairly strict criterion for sequence similarity (it is used by the functional annotation tool Blast2GO for their ‘strict’ annotation style [35]) and has been used in this literature [21], [46]. At this threshold, although hundreds or thousands of interolog inferences are correct, the fraction of correct inferences is three percent or less (see Figure 2 A and B). This small fraction is a result of the very large total numbers of predictions (between tens of thousands and two million, depending on species pair). An 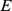-value threshold of 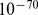 is considered strict, and has also been used in the literature [22], [46]. At this 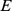-value cut-off, there are a few hundred correct inferences at most (depending on species pair) and at most 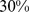 correct inferences.

We show the results for the EnsemblCompara GeneTrees homologs in Table S5 in Text S1 and those for reciprocal-best-hit homologs in Table S6 in Text S1. The number of correct predictions from *S. cerevisiae* to *S. pombe* using the manually curated set of orthologs is 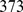, the fraction correct is 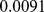 and the likelihood ratio is 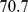. The corresponding numbers for *S. pombe* as source and *S. cerevisiae* as target species are 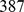, 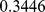, and 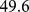. The EnsemblCompara GeneTrees homologs achieve similar fractions of correct inferences to reciprocal-hits homologs at 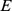-values of about 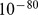; for the reciprocal-best-hit homologs, this value is about 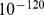.

The fraction of correct inferences depends on the coverage of the target-species interactome – note the much higher fraction of correct inferences to *S. cerevisiae* in Figure 2 B and in Tables S5 and S6 in Text S1. This is expected, and below we investigate how the fraction of correct inferences is altered when we take the coverage of the target-species interaction data set into account.

Inferences with *M. musculus* and *S. pombe* as source species achieve higher numbers of correct fractions than the inferences from other species. We hypothesise that this is due to biases in the interactomes that are particularly evident for these species. As discussed in the Introduction, such biases are present in low-throughput interaction data sets, as there is a high observed correlation between number of published papers and number of interacting partners [34]. A very large proportion of interactions in the *S. pombe* and *M. musculus* data sets come from low-throughput, hypothesis-driven studies. The observation of a homologous interaction in one species can inform experiments in another. In Figure S3 and Table S7 in Text S1, we demonstrate that in the target species, homologs of the source species are considerably more likely to interact than a randomly chosen pair of proteins. This is particularly true for *S. pombe* and *M. musculus*, presumably because of the high fraction of low-throughput data in these species interactomes. This suggests that – especially for these two species – interactions are more likely to be reported if there is a homologous interaction in another species. Evidence for the homology of protein-protein interactions will be inflated because of this effect: observed conservation rates depend both on the evolutionary conservation of interactions and on the tendency for researchers to be more likely to look for homologous interactions. Assessing the relative contributions of these two effects is hard, as they manifest in the same way (i.e. in higher observed conservation rates of interactions). Note that the likelihoods for inferences from *S. pombe* and *M. musculus* (Figure 2 C) are not large compared to the other species, as is the case with the observed fraction of correct inferences (Figure 2 B). This is because the likelihood ratio controls for some of this bias by comparing transferred interactions to random guesses between proteins that have homologs in the source-species interactome.

### Interactions conserved across species: errors in the interactome data

The bias in data-gathering discussed above leads to an overestimate in the fraction of interactions conserved, however errors in the interactome data could lead to the observed rates being underestimates. In particular, one expects the coverage of the target-species interactome to influence strongly the observed fraction of correct inferences. Previous studies left such effects of interactome incompleteness as possible explanations for the poor performance of interaction transfer on the basis of homology [21]–[23], [47]. Here we investigate the magnitude of such effects by considering several possible sources of error.

#### False positives

The effect of false positives in the *source* species leads to an *under*estimation of the fraction of interactions that are conserved, as predictions from false-positive interactions are less likely to be correct. As a simple check of the magnitude of this effect, we simulated for the three species with the largest interactomes false-positive rates in the source species in excess of 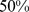 and found that the observed fractions of correct inferences are not affected greatly(see Figure S4 in Text S1).

The effect of false positives in the *target* species is the opposite of that in the source species: the fraction of interactions conserved will be *over*estimated, as some predictions will be judged to be correct by matching to a false-positive interaction in the target species. In the Materials and Methods section, we show under reasonable assumptions that this overestimation is larger than the underestimation (produced as discussed above by false positives in the source species), provided that 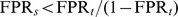, where 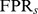 and 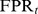 are the false-positive rates in the source and target species respectively. False-positive rates in the different species interaction sets are unlikely to be so different that this inequality fails to hold, so here we do not further consider the possibility that false positives can lead to an underestimation of the conservation of interactions.

#### Coverage of the source-species interactions

We hypothesize that the fraction of inferred interactions observed to be correct 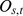 is independent of the *coverage* (which is defined as one minus the fraction of false-negatives) of the source-species interactions. The reason is as follows: although more correct inferences are observed with more interactions in the source species, more incorrect inferences are also made. We tested whether such independence held by sampling the source-species interactions (see Materials and Methods for details). The results support our hypothesis; see Figure S5 in Text S1.

#### Coverage of the target-species interactions

We hypothesised that the fraction of inferred interactions observed to be correct 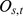 is directly (i.e. linearly) proportional to the coverage of the target-species interactions 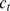. For example, if the interaction list of the target species is halved in size, then the fraction of correct inferences should also halve. We tested this hypothesis by sampling from the interaction list of the target species (see Materials and Methods) and report the mean coefficients of correlation 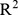 between 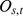 and 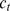: it is 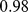 for the reciprocal-hits definition, 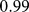 for the EnsemblCompara GeneTrees homologs, and 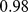 for the reciprocal-best-hits homologs. We give the full set of 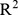 values in Tables S9, S10 and S11 in Text S1. All associated 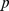-values are less than 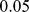.

The independence of the observed fraction of correct inferences on the source-species interaction coverage and the linear dependence on the target-species interaction coverage help motivate the following simple model for the estimated true rate of conserved interactions:
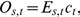
(1)where 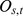 is the fraction of inferred interactions observed to be correct, 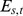 is the fraction of inferred interactions estimated to be correct (taking into account incomplete interactome coverage), and 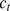 is the coverage of the target-species interactome. We emphasise that this simple model does not take into account the bias in data-gathering processes discussed above. It thus gives estimates expected with biased data; as discussed above these will be overestimates compared to estimates on data gathered at random. Due to the particularly strong bias associated with the two smallest interactomes (*S. pombe* and *M. musculus*), we estimate 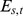 values for these species only with their most closely related species (see below). Focusing just on the four species for which there is the most interaction data, there are twelve equations (one for each pair of species, where order matters) of the form (1) for each definition of homology. As there are more unknowns than equations – only the 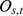 are known – one cannot solve (1) without either making some assumptions or incorporating independent estimates for values of 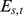 or 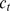. We pursue the former strategy and discuss the latter one.

We make two assumptions to calculate values of 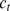, which we then use to solve for values of 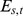. First, we assume that the *S. cerevisiae* interactome is complete (which is consistent with the literature; see Introduction and Refs. [28], [34], [48]). Altering this assumption changes all our results by a constant multiple. Second, we assume that the fraction of conserved interactions between a source species 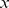 and *S. cerevisiae* is the same as from *S. cerevisiae* to species 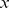; i.e. 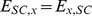. This implies that 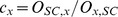. Making these assumptions allows one to decouple the 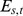 values from the 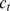 values and hence to obtain estimates for both.

We give the estimated values of 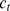 and the implied total interactome sizes in Table 3. These values lie within previous estimates (see the discussion in the Introduction and Refs. [28], [34], [48]). Our estimates of interactome size, like all others, make a series of assumptions and should therefore be taken as complementary to existing estimates. We estimate the size of the *C. elegans* and *D. melanogaster* interactomes to be larger than that of *H. sapiens*. This is surprising, as the numbers of proteins in the former two organisms are smaller (see Table 1). Homologs of *S. cerevisiae* proteins are considerably more likely than random to interact in *H. sapiens* (see Figure S3 in Text S1), which is probably due to the high proportion of interactions in *H. sapiens* that come from low-throughput studies (see Table 1). This would cause 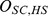 estimates to be higher than expected, and hence, via the equation 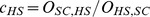, 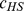 estimates would be higher than one might expect. The same effect occurs for *C. elegans*, though to a lesser extent (see Figure S3 in Text S1).

**Table 3:** Estimated interactome coverages and interactome sizes.

	reciprocal hits		EnsemblCompara GeneTrees		reciprocal best hits	
	coverage	interactome size	coverage	interactome size	coverage	interactome size
CE	0.0293 (0.0027)	256000 (24000)	0.024	310531	0.050	150742
DM	0.0707 (0.0214)	349000 (96000)	0.074	308787	0.095	240160
HS	0.1874 (0.0372)	158000 (35000)	0.162	174858	0.217	130204

We report the means and standard deviations for the reciprocal hits data over all the 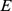-value thresholds that we investigate. These results assume that the *S. cerevisiae* interactome is complete at 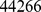 interactions.

**Figure 3:**
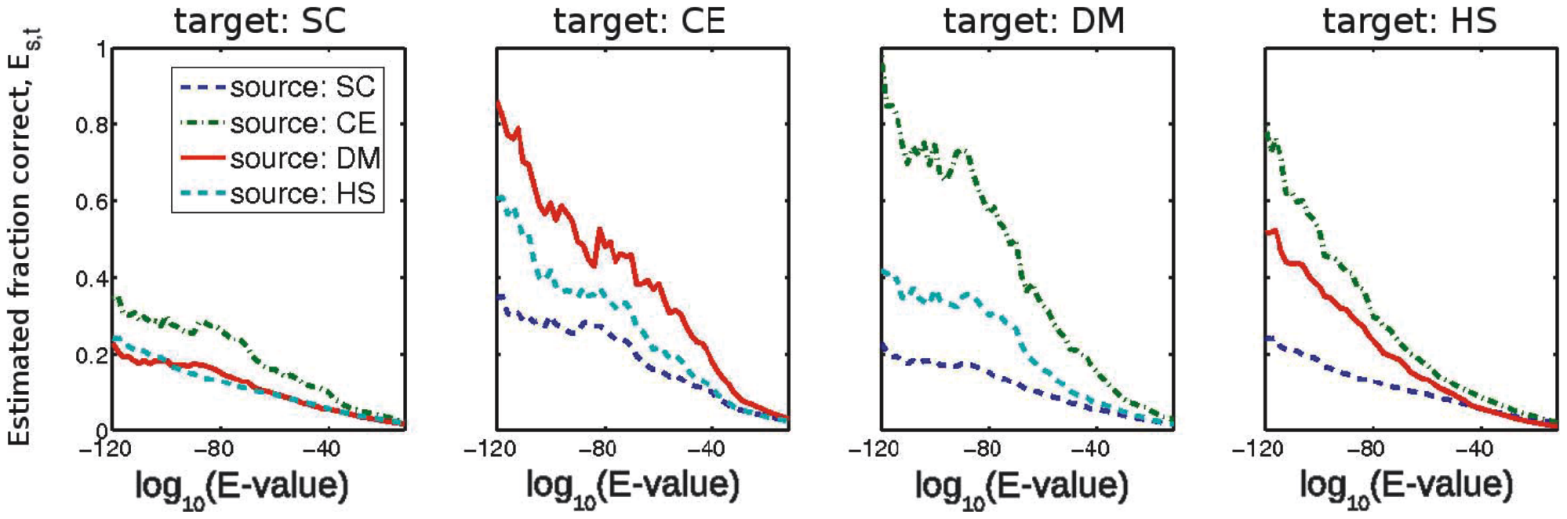
Fraction of interactions estimated to be conserved through evolution 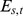, which we calculate by taking interactome coverage into account. One should expect the lower conservation rates between *S. cerevisiae* (SC) and the other species, given the known evolutionary relationships between these species. We estimate the conservation rates at 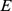-values often associated with the transfer of functional annotations (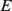-values of about 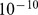) to be a few percent.

We show estimated fractions of interactions conserved in Figure 3 and Tables S12 and S13 in Text S1. As one should expect, the estimated fraction of correct inferences is lower between *S. cerevisiae* and the other three species. The estimates are highest for the most stringent definition of homology (reciprocal best hits; see Table S6 in Text S1). The extent to which strictness in definition of orthology is important for the transferability of interactions is evident from Figure 3: using reciprocal hits at 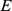-values of 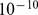 gives success rates of a few percent, even when interactome incompleteness is taken into account.

One could also solve the set of equations (1) by using independent estimates of the coverage of the interactomes 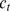. Larger estimates of 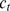 than ours would give smaller estimates of 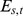. The estimated fraction of conserved interactions remains low unless one assumes very small coverages of the target-species interactome; this would imply very large total interactome sizes. For example, a 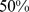 success rate for transferring interactions between *S. cerevisiae* and *H. sapiens* at an 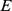-value cut-off of 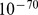 would imply an interactome size of over 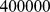 interactions for *S. cerevisiae* and over two million interactions for *H. sapiens*.

We now consider the extent of conservation between *S. cerevisiae* and *S. pombe*. Making the same assumptions as above, 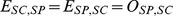, the curve shown in dashed-dotted pink in the left-most panel of Figure 2 B. We estimate 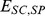 and 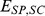 to be 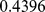 using the reciprocal-best-hits homology definition and 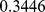 for the manually-annotated ortholog data set. The estimated fractions of interactions conserved across *S. pombe* and *S. cerevisiae*, whose last common ancestor existed about 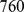 million years ago [49], are similar to those between *D. melanogaster*, *H. sapiens*, and *C. elegans*. *D. melanogaster* and *H. sapiens* shared a common ancestor about 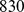 million years ago [49], and *C. elegans* shared a common ancestor with these two about 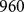 million years ago [49].

Of all of the species pairs one would expect the estimated fraction of correct inferences to be highest between *H. sapiens* and *M. musculus*, as these species shared a common ancestor about 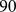 million years ago [49]. We report estimates for 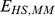 and 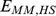 in Figure S6 in Text S1. At an 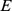-value threshold of 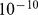, we estimate 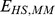 to be 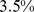 and 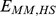 to be 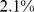. The estimated fraction correct rises above 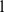 at the most stringent reciprocal-hits 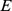-values, and is well above 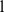 for the reciprocal-best-hits data (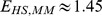 and 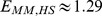) and the EnsemblCompara GeneTrees data (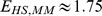 and 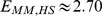). This could be because our estimates of the coverage of the two species interactomes are too low (which is equivalent to our estimates of the interactome sizes being too high). However, it is far more likely that the estimates of 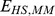 and 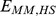 are too high because of the aforementioned biases in the data-gathering processes. Our model assumes that interactions are sampled independently in different species; however, if an interaction is known in one species, then researchers might be prompted to search for it in another. This is likely to be particularly true between *H. sapiens* and *M. musculus*.

Our estimates can be compared to the results of studies that experimentally tested for the presence of interologs. Matthews et al [21] tested predictions of inferring from *S. cerevisiae* to *C. elegans* using an orthology definition that was many-to-one (each *S. cerevisiae* was considered an ortholog of at most one *C. elegans* protein, but *C. elegans* proteins could have more than one *S. cerevisiae* ortholog). They found that between 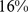 and 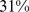 of the inferences were correct. (Compare these to our estimates for the same species pair: 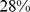 using reciprocal-best-hits data and 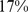 using the EnsemblCompara GeneTrees data). Using one-to-one ortholog matching, a conservation rate of between 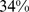 and 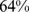 was reported between *H. sapiens* and *M. musculus* transcription factor-transcription factor interactions [24]. A recent study comparing two yeasts, *S. cerevisiae* and *Kluyveromyces waltii*, which diverged about 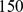 million years ago, used one-to-one orthology relationships and found that 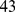 of 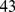 tested interactions were conserved [20].

### Interactions conserved across species: probability per million years that a duplicated interaction is lost

The results described above can be used to estimate the rate of loss of protein-protein interactions using a simple model. Assume that an interaction that existed in the last common ancestor of the source and target species has a probability 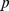 per unit time of being lost in either of the two species. For low 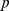, the probability that we observe an interaction between 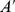 and 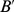 in the target species, *given that we have observed an interaction between* 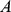 *and* 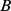 *in the source species*, is approximately 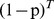, where 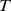 is the number of units of time since the species diverged. There are many ways to estimate 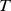, and we use the mean time and range of times given in Ref. [49].

We show how 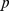 varies with the extent of sequence homology. We report results for the EnsemblCompara GeneTrees data, the reciprocal-best-hits data, and the reciprocal-hits data in windows of similarity as judged by 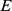-value. (i.e. 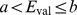 for different 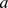 and 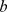). We solve the equation 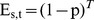 to obtain 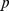.

**Figure 4:**
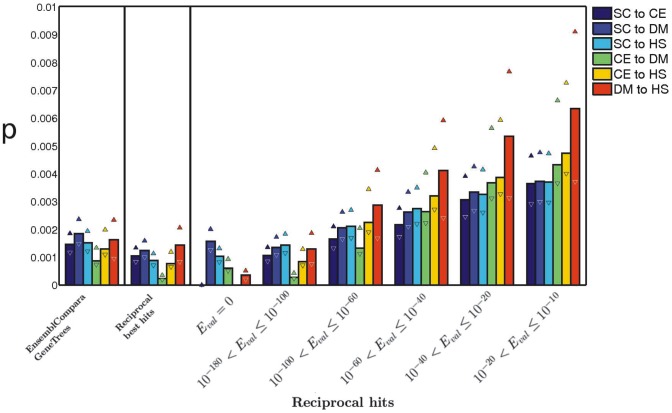
Estimates of the probability 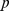 that a duplicated interaction is lost per million years. We show results for the three separate homology definitions: EnsemblCompara GeneTrees and reciprocal best hits on the left; reciprocal hits at different sequence-similarity thresholds on the right. If the proteins in two species remain highly similar in sequence, then the probability that both species retain the interaction is higher – i.e. one finds lower values of 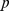 at smaller 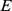-values and using the reciprocal-best-hits and EnsemblCompara GeneTrees homology relationships. The divergence time between species is needed to calculate 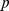; we use the estimate and range (shown in triangles) of times given in Ref. [49].

Our calculations suggest that when the divergence time of species is taken into account, the probability per million years of an interaction being lost appears to be fairly independent of species pair (see Figure 4; the indicated errors represent ranges in the estimates of 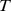). At the strictest definition of homology that we consider, we find that the rate of change of protein interactions through evolution is about 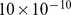 interactions lost per year. One can compare this estimate to the only other estimate we could find in the literature, which is based on a small number of experimentally tested interactions and gives an estimated rate of 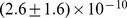 [20]. That study explicitly excludes the impact of gene duplication, so one would expect a lower rate of protein interaction change.

The step from considering the success of inferring interactions across species to inferring the rate at which interactions are lost through evolution is a large one that entails numerous assumptions and abstractions, in addition to those used to estimate values of 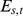. First, we suppose that the abstraction to a typical duplicated interaction is a sensible one – i.e. that it makes sense to estimate the rate at which any given duplicated interaction is lost. There are various heterogeneities in protein-protein interactions that might make this questionable. For example, genes that are duplicated might lose interactions faster than genes that are not duplicated. One response is to restrict the enquiry and seek the probability that interactions between non-duplicated genes are lost [20]. Second, we have modelled the loss of interactions as independent of each other, though whether a given interaction is lost will presumably depend on its location in the protein-protein interaction network. Indeed, we present evidence in the next section that some structural network properties can be relevant to the success of interolog inference (also see [50]). Third, we have not taken into account the role of interaction gain through evolution. Fourth, we assume that the homologs we use are in fact true paralogs or orthologs. Our estimates should be considered in light of these caveats. However, given the simplicity of our model, it is encouraging that our estimates for the rate at which interactions are lost is in broad agreement with that of Qian et al [20].

In contrast to the rate of protein sequence evolution, the rate of protein function evolution remains almost unknown [20]. Protein-protein interactions provide a window through which to view this question. Although the rate at which protein-protein interactions are lost within species has been studied [51], [52], the loss rate across species has not received much attention. Consequently, our estimates should be taken as initial ones, and we believe that they are the first ones that are based on large data sets.

### Interactions conserved across species: can one select the conserved interactions?

Given the low number of interactions transferable at stringent definitions of homology and the low success rate of transfer of interactions at less stringent definitions, we were motivated to investigate whether there are any properties that can select which inferences are likely to be correct among those made at less stringent definitions of homology (i.e. the reciprocal-hits data). Studies that use transferred interactions in building predicted sets of interactions sometimes also incorporate additional protein properties [9], [10], [12], [14]. Our intention is to investigate the extent to which certain biological properties can explain the lack of interaction conservation at less stringent definitions of homology, rather than to seek an algorithm that accurately predicts protein interactions across species. For this investigation, we focus on the three species for which there exists the most data – *S. cerevisiae*, *D. melanogaster*, and *H. sapiens* – in the hope that the results for these data sets will be influenced less by noise than the smaller data sets. Full details of the methods and results are given in the Supporting Information.

We investigate the effects of restricting inferences to those in which none of the proteins involved has more than ten homologs. We find that at lax 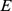-values the fraction correct is improved although the number of correct inferences is vastly reduced (Figure S7 in Text S1). We also investigate the effects of several other properties, which roughly can be divided into three classes: properties of the four proteins 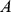, 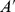, 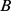 and 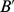 (e.g. the age of the proteins and the number of domains that make up the proteins); properties of how the interaction 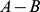 is embedded in the source-species interaction network (e.g. how many interactions the proteins have); properties of the homology relationships between 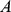 and 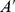 and between 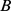 and 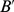 (e.g. the similarity of the lengths of proteins 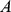 and 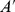). We give the list of properties that we investigate in the Supporting Information. Although many of the properties that we consider do indeed help select interactions that are more likely to be conserved, we find that they only do so with minimal efficacy, as each property helps to make an inference no more than 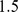 times more likely (see Figures S8 and S9 in Text S1). These results suggest that at 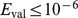, the observed fraction deemed correct from *H. sapiens* to *S. cerevisiae* could increase from 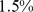 to 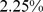 (with the number of correct inferences reduced by 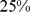).

### Interactions conserved within species: success of ‘one-same’ and ‘both-different’ inferences

We now examine the evidence for the homology of protein-protein interactions within a species. Our principal aim is to compare this evidence to that for across-species inferences.

We consider three sets of inferences in the within species case: one-same, both-different-1 and both-different-2 (see Figure 1).

One-same inferences are inferences of 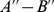 from 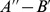. The both-different-1 class excludes all inferences of the one-same type. In the both-different-2 class we identify the closest homologous interaction for every 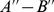. That is, we keep only the closest interaction and then remove all one-same inferences from the list. In order to identify this closest interaction we order the possible inference pairs by their maximum blastp 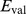. For example, suppose that 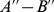 can be inferred from both 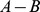 and 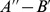, at a given homology cut-off. The 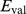 for the inference from 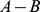 will be the larger of 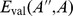 and 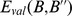 whilst that for the inference 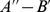 will be 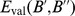. Thus, for the inference from 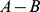 to be considered closer than that from 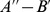, both 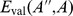 and 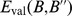 must be lower than 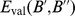. This means that for some interactions at a given homology cut-off an inference will be made by both-different-1 but not by both-different-2. The both-different-2 set is designed to completely remove the effects of one-same inferences. It operates under the premise that the presence of the predicted interaction (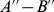) is most parsimoniously explained by the evolutionarily closest interaction.

**Figure 5:**
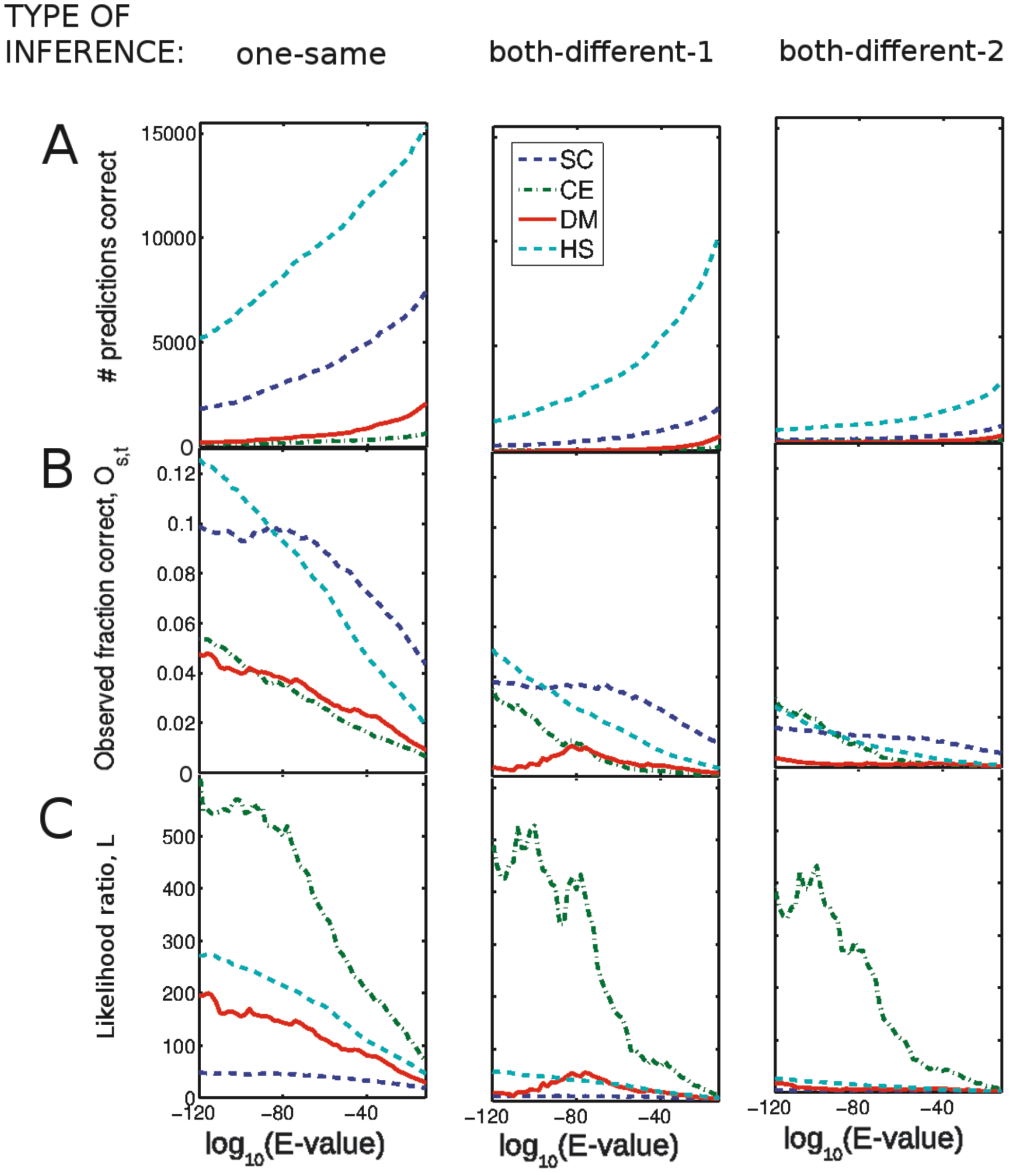
Inferences within a species: ‘one-same’ inferences (left) dominate ‘both-different-1’ inferences (centre) and both-different-2 inferences (right)). For inferences within *S. cerevisiae* (SC), *C. elegans* (CE), *D. melanogaster* (DM), and *H. sapiens* (HS), one-same inferences dominate for (A) the number of correct inferences, (B) the fraction of inferences observed to be correct 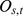, and (C) the likelihood 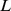 that the inferences are correct.The very large likelihoods for *C. elegans*, particularly for the both-different cases, are due to small-number effects.

We conduct an investigation for our various within-species inferences similar to the across-species case. See Figure 5 and Table 4; additionally we provide a version of Figure 5 for percentage sequence identity instead of 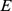-value in Figure S10 in Text S1. The number of correct one-same interactions is large in comparison to both across-species interactions and to both-different-1 interactions. Indeed, one-same interactions represent a sizeable fraction of the aggregate interaction lists (compare Figure 5 A and Table 1). However, a comparison to Figure 2 shows that the observed fraction of correct one-same inferences is comparable to and sometimes lower than that for across-species inferences (depending on the species pair).

**Table 4:** Within species: Ratio of correct inferences using the real data compared to randomly rewired interactions.

	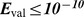			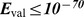		
	One-same	Both-different-1	Both-different-2	One-same	Both-different-1	Both-different-2
SC	4.6 (0.48)	2.2 (0.065)	1.5 (0.26)	7.9 (0.99)	3.7 (0.62)	2.4 (0.71)
CE	3.3 (0.26)	1.5 (0.10)	1.5 (0.25)	8.2 (0.61)	15 (8.5)	7.4 (0.54)
DM	3.5 (0.38)	1.5 (0.084)	1.1 (0.14)	11 (2.2)	15 (9.6)	2.4 (2.0)
HS	4.9 (0.33)	2.0 (0.025)	1.1 (0.14)	10 (0.95)	4.6 (0.33)	2.8 (0.53)

The one-same inferences perform better than the both-different inferences. The values in this table should be compared to those in Table 2. A comparison with Figure 5 C illustrates that the choice of how to measure the improvement over random can have very large effects on the results.

The both-different-1 results show that within-species inferences have a similar success rate to that of across-species inferences. This is different from the result of Mika and Rost [23] who found that within-species interactions (with one-same inferences removed) were more successful. The reason for this difference is unclear, however there are four major differences between our methodology for both-different-1 and that used by Mika and Rost (Figure S11 in Text S1). Firstly, unlike Mika and Rost, we do not separate different data sets. This is unlikely to be the cause of the difference as Mika and Rost state that the same trends are observed even if they do not carry out this procedure. Secondly, we use blastp 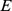-value as our indicator of protein homology as opposed to HVAL. Mika and Rost claimed that HVAL is a better method for the identification of homologs, and it is certainly true that blastp 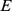-value is not the best homolog indicator. However, it is by far the most widely used measure of homology. If HVAL is a better homolog indicator this should just mean that all our results are slightly worse than those of Mika and Rost. Thirdly, there is a change in database size. In this study we use over 44,000 interactions for *S. cerevisiae*, whereas Mika and Rost used just under 6000 of them. In predicting within-species interactions for *S. cerevisiae* their number of true positives is approximately 180 at their laxest HVAL cut-off of 0. This compares to nearly 1800 for us at our laxest E-value cut off and around 250 at our strictest. Fourthly, the majority of Mika and Rost's conclusions use multiple species data for across-species interaction inference. The use of multiple species affects the accuracy in a specific way. The ability to infer interactions is described as the ratio of correct inferences to the number of incorrect inferences. If multiple species data is used the number of correct inferences will increase more slowly than the number of incorrect inferences as the correct inferences from each species to the target species tend to repeat more than the incorrect inferences. This means the ratio of correct inferences to incorrect inferences will decrease as the number of species we infer from increases. However, Mika and Rost also report a difference using only pairs of species. Thus none of these methodological or data differences provide an obvious explanation of why different conclusions are reached.

In our both-different-2 results (See: Table 5, Figure 5), in which we remove one-same interactions along even more stringent criteria we observe that across-species inferences appear to be more successful than within-species.

**Table 5:** Fraction of observed correct inferences 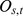 at blastp 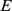-value cut-offs of 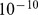 and 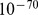 for across-species and both-different-1 within-species transferred interactions.

Fraction of correct inferences, 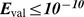					
target species		SC	CE	DM	HS
source species	SC	0.0128(0.0055)	0.0006	0.0018	0.0041
	CE	0.0207	0.0004 (0.0002)	0.0029	0.0041
	DM	0.0157	0.0007	0.0012 (0.0006)	0.0024
	HS	0.0175	0.0006	0.0017	0.0029 (0.0009)

The numbers in brackets give results for the both-different-2 inferences. The data show that within-species inferences are not always more accurate than across-species inferences.

### Concluding remarks

Using six species, a mixture of low-throughput and high-throughput binary protein-protein interaction data and three different sets of homology definitions, we have investigated the conservation of interactions across and within species. Several factors mean that observed conservation rates do not reflect true evolutionary conservation rates. The biases in the data suggest that observed conservation rates will be inflated due to preferential investigation of homologous interactions. We develop a framework that takes interaction incompleteness into account – in contrast to previous studies, which have side-stepped the question of interactome errors. Using this framework, we are able to estimate interactome sizes with a method that is different from others in the literature.

Our estimates for the fraction of conserved interactions are very low for definitions of homology that are often associated with the transfer of functional annotations across species. We emphasise that our results will be *over*estimates due to the preferential investigation of homologous proteins in multiple species.

We used our results on the conservation of interactions to estimate the rate at which protein-protein interactions are lost through evolution, though we stress the caveats involved with such an estimate. Given that inferred interactions are not accurate unless stringent definitions of homology are used, but that few interactions are transferable when such definitions are in place, we considered the possibility that certain types of inference were substantially less likely to yield conserved interactions. For example, we considered it possible that inferences from proteins in large protein families were substantially less accurate. Despite investigating a range of properties that might influence the conservation of interactions, we found no properties that gave much improvement in conservation rates when taken into account.

The present study concentrates on the success of interolog inferences, which is the basis for a large number of widely-used methods to predict interactions [6]–[17]. We urge extreme caution in interpreting interactions transferred across species unless the definition of homology employed is a strict one, and we believe that interactome incompleteness is not solely responsible for the lack of observed conservation of interactions.

## Materials and Methods

### Protein-protein interaction data

Several publicly available databases gather interaction data from multiple sources [53]–[60]. We assembled our interaction lists from four of the largest databases: BioGRID (www.thebiogrid.org [58]; downloaded in June 2010), IntAct (www.ebi.ac.uk/intact [53]; downloaded in June 2010), MINT (mint.bio.uniroma2.it/mint [56]; downloaded in June 2010), and HPRD (hprd.org [60]; downloaded in July 2010). We use a locus-based approach; in other words, we consider only one protein isoform per gene and achieve this by mapping all protein identifiers to the identifiers used in STRING [57].

From these databases we select only direct protein-protein interaction data, thereby excluding all indirect association data, such as from tandem affinity purification experiments. We used interactions with ‘physical association’ evidence type from the IntAct database; ‘biophysical’ or ‘protein complementation’ assay type from the MINT database; ‘reconstituted complex’, ‘PCA’, ‘Co-crystal structure’ or ‘yeast-two-hybrid’ from the BioGRID database; and all interactions from the HPRD, as it only contains binary interaction data.

### Homology data

We downloaded amino acid sequences for the proteins of the species considered from the NCBI (ftp://ftp.ncbi.nih.gov/refseq/release). We ran blastp using default parameters (except for setting the maximum number of hits retrieved to be 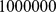 and the 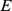-value cut-off to be 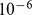). For each query, we selected the hit with the lowest 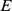-value and only kept pairs that were found as ‘query-hit’ and as ‘hit-query’ (‘reciprocal hits’). Our homology relationships are thus many-to-many. In Table S2 in Text S1 we give the numbers of reciprocal hits found at two different similarity cut-offs.

We also consider only reciprocal best hits, in which two sequences are considered each others' reciprocal best hits if the first is the best hit when the second is queried against the database and the second is the best hit when the first is queried against the database. The reciprocal-best-hit criterion gives one-to-one query-hit matches. We also require that both hit-query and query-hit 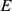-values must be 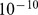 or lower. We give the numbers of reciprocal-best-hit matches in Table S3 in Text S1. The reciprocal best hits method suffers from being dependent on the precise database used for the queries. There is also no guarantee that the closest-sequence homolog is the closest functional homolog.

We additionally consider homologs as defined by EnsemblCompara GeneTrees [4]. This method is based on the inference of multiple potential gene tree topologies; it penalises those topologies which are inconsistent with known species relationships.

### Comparisons to random: likelihood measure

Following the work of Jansen et al [61] and Yu et al [22], we consider the likelihood ratio 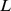 for an interolog inference (from interacting proteins 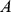 and 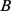 to an interaction between their homologs 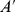 and 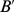) to be a true prediction. The likelihood, which is a function of the source species and target-species interaction data (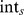 and 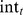), relates the odds of finding a conserved interaction (a *positive*) before and after knowing the interaction data:
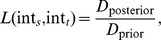
where 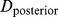, which denotes the odds of finding a positive (i.e. the ratio of the probability of finding a positive to that of finding a negative) *after* we have inferred interactions, is given by
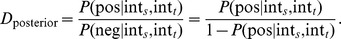
The quantity 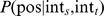 is the probability of finding a positive after we have considered the interaction data 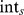 and 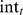. This quantity is the observed fraction of correct inferences 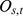. The quantity 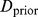, the prior odds of finding a positive in the target species given that there exist homologs of both proteins in the source-species interactome, is given by
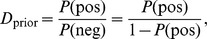
where 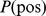 gives the number of correct inferences among all possible inferences *before* we consider the interaction data (but assuming that we know which proteins are in the source-species interactome). The quantity 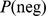 is the same except for a negative. The number of possible inferences is equal to every pair of proteins in the target species, each of which have a homolog in the source species interactome. If there are 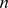 proteins in the target species with homologs in the source species interactome, then this is 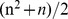 (including self-interactions). In the one-same case (inferring from an interaction between 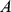 and 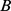 to one between 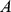 and 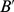), one can make inferences to any pair of proteins as long as one of them is in the interactome and the other has a homolog somewhere else in the interactome. The number of possible correct inferences is the number of interactions 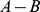 in the target species for which both 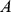 and 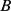 have homologs in the source species interactome.

Predictions are more likely to be true for higher values of the likelihood ratio 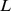. A likelihood of 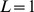 designates that prediction is no better than guessing that there is an interaction between any pair of proteins in the target species, provided both of them have homologs in the source species interactome.

### Comparisons to random: rewiring

We randomised the interactions from which we were inferring by rewiring them, such that the number of interacting partners of each protein is kept constant. By keeping constant the number of times each protein appears in the interaction list, we ensure that differences we identify are due to the interactions themselves rather than to the properties of the proteins. We perform this rewiring of the source-species interactions ten times for each species pair.

### Considering false positives

One can estimate the magnitude of underestimation from false positives in the source species by assuming that false positives and true positives contribute in a linear fashion to the aggregate fraction of correct inferences:
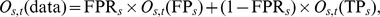
where 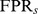 is the false-positive rate in the source species; and 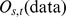, 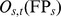, and 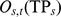 are, respectively, the fraction of correct inferences observed for the data, the fraction that would be observed with 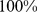 false-positive source-species interactions, and the fraction that would be observed with 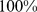 true-positive source-species interactions. The largest possible underestimation arises with 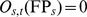. The largest underestimation is thus
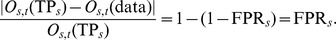



Assuming that whether or not an interaction is a false positive and whether or not it is predicted as an inferred interaction are independent assumptions, it follows that the fraction of inferences that are falsely considered to be correct is simply the false-positive rate of the target-species interactions:
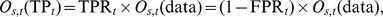
where 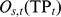 is the fraction of correct inferences that would be observed if all of the target species data were true-positives, and 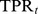 and 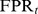 are the true- and false-positive rates in the target species. The overestimation caused by false positives in the target species is thus
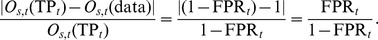



Under these assumptions, and provided that 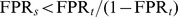, the underestimation caused by false positives in the source species is always less than the overestimation caused by the target species.

### Simulating false negatives by sampling

To simulate the effect of false negatives, we sub-sample from the interaction lists by randomly selecting 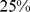, 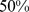, and 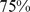 of the interactions. At each of these values, we make ten random samplings.

## Supporting Information

Text S1 Supplementary material available including figures and tables supporting the results described in the paper and a description of the tests carried out for the selection of conserved interactions.
(PDF)
