# Supplementary material for: What Evidence Is There for the Homology of Protein-Protein Interactions?
Source: PLoS Comput Biol. 2012 Sep 20;8(9):e1002645. doi: 10.1371/journal.pcbi.1002645 (PMC3447968; doi:10.1371/journal.pcbi.1002645)
Supplement: Text S1 — Supplementary material available including figures and tables supporting the results described in the paper and a description of the tests carried out for the selection of conserved interactions. (PDF) [file pcbi.1002645.s001.pdf]

Supporting Information: What Evidence is There for the Homology of Protein-Protein Interactions?

Anna C. F. Lewis, Nick S. Jones, Mason A. Porter, Charlotte M. Deane

Supplementary text for the section 'Interactions conserved across species: can one select the conserved interactions?"

We are considering the transfer of interactions between interacting proteins A and B in a source species to proteins A' and B' in a target species, where A and A' are homologs and B and B' are homologs. For any given inferred interaction in the target species, there can be multiple possible interactions in the source species from which it could have been inferred. In order to consider properties of the proteins in the source species, it is necessary to state which of these multiple possible interactions is considered to underlie a given inferred interaction $A' - B'$ in the target species. We select, as the 'closest' inference, the one that would be made using the strictest definition of homology (i.e., the one with the minimum value of $\max\{E_{\text{val}}(A, A'), E_{\text{val}}(B, B')\}$).

The first property that we investigated was the size of the family to which a protein belongs. If only one or a few interactions between proteins from one family and proteins from another family is needed for the maintenance of biological function, then one might expect that an inference from or to proteins with many homologs in the other species would be less conserved. We tested how inferences to and from proteins in large protein families affected our results by discarding all predictions in which any of proteins A, B, A', and B' had more than 10 homologs in the other species. This definition of size of family is clearly dependent on the E-value threshold, as a protein's family size becomes smaller at stricter E-values. Our intention was to get an idea of the magnitude of the effect of large families, so we chose one definition of a large protein family (i.e., those of size at least 10). We show the results in Figure S7.

We also investigated the effects of several other properties, listed below; the list is by no means exhaustive. We choose to investigate the utility of these properties for selecting conserved interactions by defining the following ratio: the $O_{s,t}$ values obtained using only the inferences with values above or below the median value of the property of interest to the values obtained with all inferences.

In inferring $A' - B'$ from $A - B$, we assess the relevance of the following properties:

- The product of the number of homologs of A in the target species and the number of homologs of B in the target species (where homologs are defined as above).
- The product of the number of homologs of A' in the source species and the number of homologs of B' in the source species.
- The total number of inferences to the interaction $A' - B'$.
- The difference in the ages of A and B. As a proxy for protein age, we use 'excess retention' (ER) [1], which counts the number of species in which a protein has orthologs. (We use the Inparanoid database to define orthologs [2].) We were prompted to investigate this property by Refs. [3, 4].
- The difference in the ages of A' and B'.
- The sum of the ages of A and B.
- The sum of the ages of A' and B'.
- The product of the number of domains of A and the number of domains of B. We defined domains via SCOP (Structural Classification of Proteins [5]).
- The product of the number of domains of A' and the number of domains of B'.

- The geodesic edge betweenness centrality of the interaction between A and B [6]. Roughly, this centrality is given by the number of shortest paths between pairs of proteins that pass through the interaction in question.
- The number of triangles in which $A - B$ participates as a fraction of the triangles in which it could participate. This quantity, called the 'matching index' in Ref. [7], gives a measure of local clustering.
- The product of the number of interacting partners of A with the number of interacting partners of B divided by the total number of interactions.
- $\min\{E_{\mathrm{val}}(A, A'), E_{\mathrm{val}}(B, B')\}$
- $E_{\mathrm{val}}(A, A') \times E_{\mathrm{val}}(B, B')$
- $\mathrm{pid}(A, A') + \mathrm{pid}(B, B')$
- $\mathrm{pid}(A, A') \times \mathrm{pid}(B, B')$
- $g(A, A') + g(B, B')$
- $\mathrm{ac}(A, A') + \mathrm{ac}(B, B')$
- $\mathrm{ls}(A, A') + \mathrm{ls}(B, B')$,

where pid is the percentage sequence similarity over the aligned region, g is the number of gaps in the sequence alignment, the alignment coverage ac is the minimum of the fraction of the query covered by the alignment and the fraction of the hit covered by the alignment, and the length similarity ls is the length of the shorter sequence divided by that of the longer sequence.

We show the results in Figures S8 and S9.

References

1. Saeed R, Deane CM (2006) Protein protein interactions, evolutionary rate, abundance and age. BMC Bioinformatics 7: 128.
2. O'Brien KP, Remm M, Sonnhammer ELL (2005) Inparanoid: a comprehensive database of eukaryotic orthologs. Nucleic Acids Research 33: D476–D480.
3. Qin H, Lu HHS, Wu WB, Li WH (2003) Evolution of the yeast protein interaction network. Proceedings of the National Academy of Sciences 100: 12820–12824.
4. Kim WK, Marcotte EM (2008) Age-dependent evolution of the yeast protein interaction network suggests a limited role of gene duplication and divergence. PLoS Comput Biol 4: e1000232.
5. Murzin A, Brenner S, Hubbard T, Chothia C (1995) SCOP: a structural classification of proteins database for the investigation of sequences and structures. Journal of Molecular Biology 247: 536–540.
6. Newman MEJ (2010) Networks: an introduction. Oxford University Press.
7. Ascoli GE (2003) Computational Neuroanatomy – Principles and Methods. Humana Press.

Table S1: The fraction of interactions for a given species that have supporting evidence in publications that report fewer than N interactions. *M. musculus* (MM) and *S. pombe* (SP) have a very high proportion of interactions that are supported by low-throughput publications. The fraction of reported interactions from low-throughput studies for *H. sapiens* is also high. These trends remain the same for the range of N we investigate here. Note that some interactions may have support from publications that have not been annotated by a pubmed ID in one of the databases, so these numbers may be underestimates of the fraction of interactions with support from low-throughput studies (note that this is why a small fraction of the *S. pombe* interactions are not supported by evidence from publications that report 100 or fewer interactions.)

N	SC	CE	DM	HS	MM	SP
20	0.11	0.13	0.06	0.56	0.62	0.56
50	0.13	0.13	0.07	0.59	0.75	0.79
100	0.15	0.15	0.07	0.61	0.82	0.97
200	0.16	0.22	0.07	0.63	0.84	0.97
500	0.18	0.22	0.07	0.66	0.85	0.97

Table S2: Reciprocal-hits homology relationships at two different E-value thresholds.

**Number of homology relationships, $E_{\text{val}} \leq 10^{-10}$**							
target species		SC	CE	DM	HS	MM	SP
source species	SC	9752	15427	20373	34988	31443	16327
	CE	15427	103265	47023	78067	70543	17919
	DM	20373	47023	51434	149693	134237	22749
	HS	34988	78067	149693	217629	557652	41976
	MM	31443	70543	134237	557652	495248	40304
	SP	16327	17919	22749	41976	40304	6577
**Number of proteins involved in homology relationships, $E_{\text{val}} \leq 10^{-10}$**							
source species	SC	2446	2062	2428	2547	2435	3561
	CE	2516	9233	5374	5441	5309	3055
	DM	3362	5658	6366	7138	6914	4007
	HS	4428	7728	9435	10229	12671	5811
	MM	4211	7320	8913	13264	10756	5616
	SP	3260	2191	2619	2837	2815	1903

**Number of homology relationships, $E_{\text{val}} \leq 10^{-70}$**							
source species	SC	3349	1085	1448	1961	1795	2515
	CE	1085	8669	3294	4320	3924	1252
	DM	1448	3294	3702	7546	6714	1721
	HS	1961	4320	7546	32581	77188	2553
	MM	1795	3924	6714	77188	62405	2434
	SP	2515	1252	1721	2553	2434	791
**Number of proteins involved in homology relationships, $E_{\text{val}} \leq 10^{-70}$**							
source species	SC	1202	473	687	741	683	1479
	CE	525	4284	1527	1585	1484	669
	DM	757	1610	2350	2904	2720	977
	HS	988	2116	3903	5882	10143	1376
	MM	912	1893	3467	10536	6196	1321
	SP	1359	586	820	922	911	763

Table S3: Number of reciprocal-best-hits homology relationships. As this is a one-to-one orthology definition, the number of homology relationships and the number of proteins involved in homology relationships are the same.

target species		SC	CE	DM	HS	MM	SP
source species	SC	-	1230	1626	1749	1746	2666
	CE	1230	-	2761	2886	2875	1477
	DM	1626	2761	-	4347	4332	2037
	HS	1749	2886	4347	-	12579	2225
	MM	1746	2875	4332	12579	-	2205
	SP	2666	1477	2037	2225	2205	-

Table S4: Homology relationships as defined by EnsemblCompara GeneTrees.

**Number of homology relationships**						
target species		SC	CE	DM	HS	MM
source species	SC	-	5276	5109	6170	4943
	CE	5276	-	13334	12656	10210
	DM	5109	13334	-	12465	10196
	HS	6170	12656	12465	-	16227
	MM	4943	10210	10196	16227	-
**Number of proteins involved in homology relationships**						
source species	SC	-	2315	2346	2456	2363
	CE	3589	-	5623	5645	5501
	DM	3514	5945	-	6189	5903
	HS	4119	7577	7822	-	12461
	MM	3812	7006	7252	12807	-

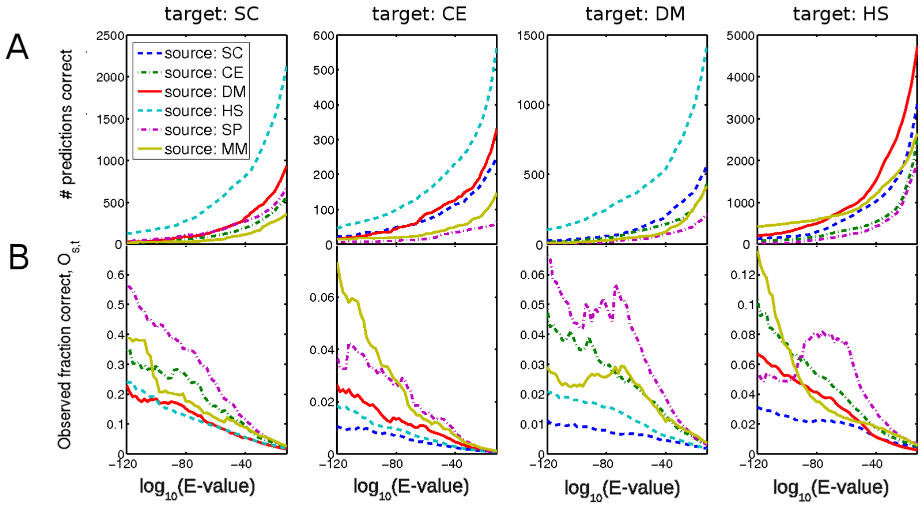

Figure S1: As for Figure 2 A and B of the main text, but with different scales for the *y*-axes. We show the results of inferring interactions from *S. cerevisiae* (SC), *C. elegans* (CE), *D. melanogaster* (DM), *H. sapiens* (HS), *S. Pombe* (SP), and *M. musculus* (MM) to the first four of those species. (A) Number of correct interolog inferences across species, for different `blastp` E-value cut-offs. (B) Fraction of all inferences that are observed in the interactions of the target species, $O_{s,t}$.

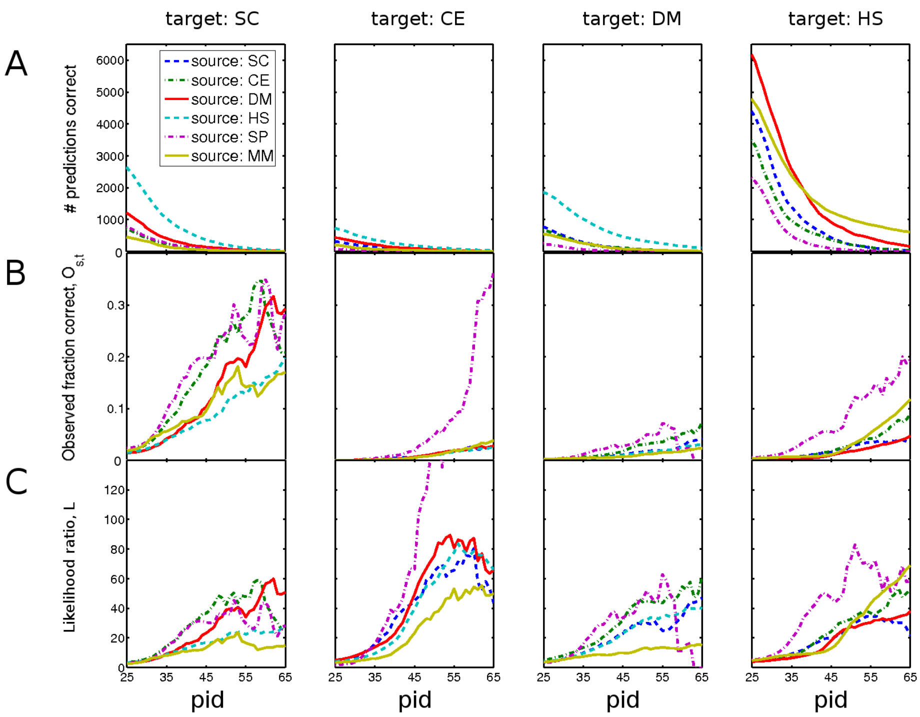

Figure S2: As for Figure 2 of the main text, but using thresholds of percentage sequence identity (pid) rather than thresholds on E-value. We show the results of inferring interactions from *S. cerevisiae* (SC), *C. elegans* (CE), *D. melanogaster* (DM), *H. sapiens* (HS), *S, Pombe* (SP), and *M. musculus* (MM) to the first four of those species. (A) Number of correct interolog inferences across species, for different `blastp` E-value cut-offs. (B) Fraction of all inferences that are observed in the interactions of the target species, $O_{s,t}$. (C) The likelihood ratio L that an inference is correct.

Table S5: Across species inferences using the EnsemblCompara GeneTrees data. These results show the same quantities as for Figure 2 and Table 2 of the main text.

**Number of correct inferences**					
target species		SC	CE	DM	HS
source species	SC	-	197	349	1601
	CE	203	-	146	421
	DM	338	137	-	841
	HS	1197	265	528	-
	MM	112	55	89	532
**Fraction of correct inferences** $O_{s,t}$					
source species	SC	-	0.004	0.008	0.025
	CE	0.166	-	0.031	0.047
	DM	0.101	0.013	-	0.042
	HS	0.153	0.013	0.025	-
	MM	0.280	0.042	0.060	0.283
**Likelihood ratio** L **that an inference is correct**					
source species	SC	-	28.9	18.8	31.6
	CE	25.8	-	43.8	42.3
	DM	19.5	66.8	-	53.2
	HS	30.7	55.3	50.7	-
	MM	33.0	62.9	48.2	114
**Comparison to rewired source-species interactions**					
source species	SC	-	19 (11)	13 (2.3)	15 (1.5)
	CE	12 (2.4)	-	27 (10)	24 (16)
	DM	11 (2.4)	32 (18)	-	18 (1.6)
	HS	12 (1.4)	14 (3)	13 (0.93)	-
	MM	18 (7.9)	31 (21)	20 (11)	36 (11)

Table S6: Across species inferences using the reciprocal-best-hits data. These results show the same quantities as for Figure 2 and Table 2 of the main text.

**Number of correct inferences**					
target species		SC	CE	DM	HS
source species	SC	-	106	166	668
	CE	106	-	106	166
	DM	166	106	-	290
	HS	668	166	290	-
	MM	59	26	37	606
	SP	222	20	26	133
**Fraction of correct inferences** $O_{s,t}$					
source species	SC	-	0.014	0.020	0.073
	CE	0.275	-	0.076	0.103
	DM	0.214	0.033	-	0.066
	HS	0.335	0.031	0.044	-
	MM	0.488	0.080	0.091	0.281
	SP	0.440	0.062	0.071	0.299
**Likelihood ratio** L **that an inference is correct**					
source species	SC	-	51.3	39.8	69.0
	CE	41.6	-	78.8	66.5
	DM	42.4	113	-	69.6
	HS	76.1	107.6	77.5	-
	MM	55.9	72.7	67.2	107
	SP	62.0	50.8	45.3	73.5
**Comparison to rewired source-species interactions**					
source species	SC	-	38 (29)	23 (6.0)	26 (8.8)
	CE	18 (6.4)	-	26 (10)	25 (11)
	DM	26 (7.8)	55 (37)	-	28 (9.5)
	HS	20 (3.4)	24 (8.4)	23 (6.7)	-
	MM	16 (6.8)	21 (0.36)	30 (0.62)	34 (16)
	SP	21 (4.7)	11 (6.6)	18 (6.9)	41 (33)

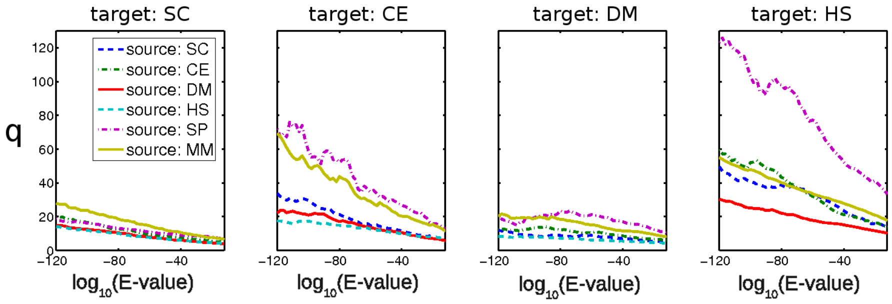

Figure S3: Proteins in the target species that have homologs in the source-species interactome are q times more likely to interact than a pair chosen uniformly at random from the target-species interactome. This ratio is the quantity $P(\text{pos})$ (defined in Materials and Methods) divided by the density of interactions in the target-species interactome. This indicates a bias such that proteins that have been investigated for protein-protein interactions in one species are not independent of those that have been investigated in another. This is particularly true for *S. pombe* (SP) and *M. musculus* (MM).

Table S7: As for Figure S3, but for the EnsemblCompara GeneTrees data. The density of interactions between proteins in the target species that have homologs in the source species divided by the density of interactions in the target-species interactome.

target species		SC	CE	DM	HS
source species	SC	-	3.53	1.60	6.13
	CE	7.06	-	3.02	8.78
	DM	5.31	5.17	-	6.21
	HS	5.39	6.26	2.04	-
	MM	10.8	17.9	5.30	26.2

Table S8: As for Figure S3, but for the reciprocal-best-hits data. The density of interactions between proteins in the target species that have homologs in the source species divided by the density of interactions in the target-species interactome.

target species		SC	CE	DM	HS
source species	SC	-	6.86	2.09	8.65
	CE	8.34	-	4.18	13.2
	DM	5.90	7.75	-	7.70
	HS	6.07	7.65	2.40	-
	MM	15.5	30.4	6.01	27.7
	SP	11.5	33.1	6.82	43.8

Table S9: Results of tests carried out to examine the hypothesis that the observed fraction of correct inferences $O_{s,t}$ is directly proportional to the coverage of the target-species interactions c_t for the reciprocal-hits data. As described in Materials and Methods, we sub-sampled from the target-species interactome 10 times by selecting a fraction f of the target-species interactions. We investigated $f = 0.25$, $f = 0.5$, and $f = 0.75$. For each of the 10 experiments, we calculated the coefficient of correlation R^2 between $O_{s,t}$ and c_t at these three values of f and also for $f = 1$ (i.e. the complete data set). Here we report the means and standard deviations of the results of the 10 experiments. All the results have an associated p-value of less than 0.05 across all E-value thresholds tested. We show the results at two different E-value thresholds: 10^{-10} and 10^{-70}.

$E_{\text{val}} \leq 10^{-10}$					
target species		SC	CE	DM	HS
source species	SC	-	0.9970 (0.0020)	0.9980 (0.0020)	0.9998 (0.0002)
	CE	0.9976 (0.0030)	-	0.9980 (0.0029)	0.9996 (0.0003)
	DM	0.9989 (0.0012)	0.9970 (0.0032)	-	0.9998 (0.0001)
	HS	0.9996 (0.0006)	0.9981 (0.0024)	0.9993 (0.0009)	-
	MM	0.9982 (0.0016)	0.9898 (0.0104)	0.9971 (0.0021)	0.9995 (0.0004)
	SP	0.9987 (0.0009)	0.9814 (0.0149)	0.9959 (0.0034)	0.9993 (0.0007)
$E_{\text{val}} \leq 10^{-70}$					
source species	SC	-	0.9865 (0.0098)	0.9757 (0.0227)	0.9963 (0.0039)
	CE	0.9864 (0.0190)	-	0.9845 (0.0143)	0.9966 (0.0042)
	DM	0.9879 (0.0155)	0.9753 (0.0268)	-	0.9986 (0.0015)
	HS	0.9971 (0.0025)	0.9929 (0.0071)	0.9953 (0.0032)	-
	MM	0.9819 (0.0146)	0.9397 (0.0848)	0.9687 (0.0339)	0.9982 (0.0012)
	SP	0.9900 (0.0083)	0.9265 (0.0716)	0.9627 (0.0283)	0.9930 (0.0066)

Table S10: As for Table S9, but for the EnsemblCompara GeneTrees data. The means and standard deviations of the coefficient of correlation R^2 between $O_{s,t}$ and c_t . All the results have an associated p-value of less than 0.05.

target species		SC	CE	DM	HS
source species	SC	-	0.9928 (0.0067)	0.9973 (0.0019)	0.9993 (0.0006)
	CE	0.9939 (0.0048)	-	0.9918 (0.0088)	0.9973 (0.0022)
	DM	0.9966 (0.0034)	0.9896 (0.0115)	-	0.9985 (0.0012)
	HS	0.9990 (0.0014)	0.9951 (0.0037)	0.9978 (0.0026)	-
	MM	0.9867 (0.0099)	0.9804 (0.0221)	0.9831 (0.0173)	0.9985 (0.0018)

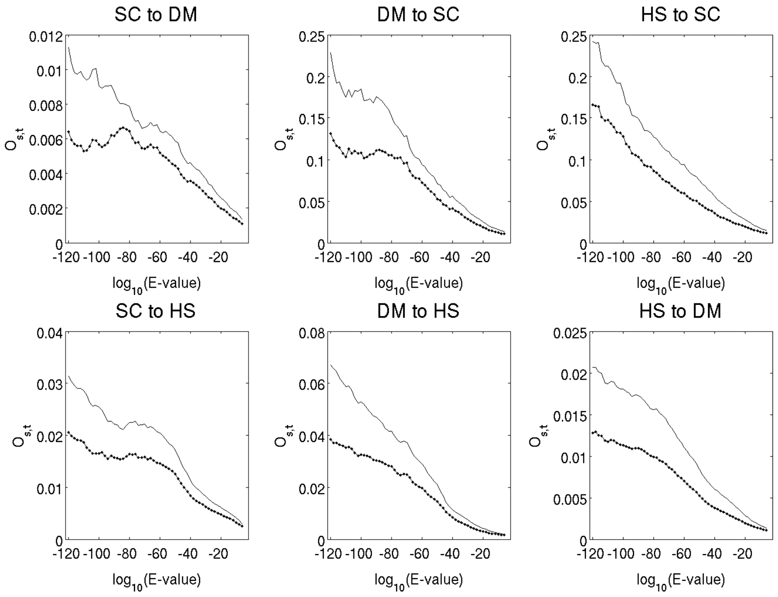

Figure S4: Even rewiring half of the source-species interactions does not have a large influence on the observed fraction of correct inferences $O_{s,t}$. To simulate the effect of false positives in the source-species interactions, we randomly rewire half of them (see Materials and Methods). We show results for the actual data (solid curve) and the mean of 10 sets of rewired data (joined-up-dotted curve). The rewiring process simulates a false-positive rate of $(50 + h/2)\%$, where h is the false-positive rate in the data. One can compare the observed fraction of correct inferences for the actual and rewired data to obtain a rough indication of how much the fraction deemed to be correct would differ if the false-positive rate were 0%. We found across the full range of E_{val} thresholds that rewiring half of the data had little impact on the fraction of inferences that were correct. Note that, as discussed in the main text, although false positives in the source species lead to an underestimation of the fraction of correct inferences, false positives in the target species lead to *over*estimation of the fraction of correct inferences.

Table S11: As for Table S9, but for the reciprocal-best-hits data. The means and standard deviations of the coefficient of correlation R^2 between $O_{s,t}$ and c_t . All the results have an associated p-value of less than 0.05.

target species		SC	CE	DM	HS
source species	SC	-	0.9919 (0.0078)	0.9926 (0.0108)	0.9983 (0.0012)
	CE	0.9871 (0.0097)	-	0.9887 (0.0131)	0.9938 (0.0056)
	DM	0.9942 (0.0063)	0.9901 (0.0080)	-	0.9968 (0.0026)
	HS	0.9977 (0.0015)	0.9934 (0.0035)	0.9959 (0.0033)	-
	MM	0.9838 (0.0157)	0.9339 (0.0662)	0.9611 (0.0338)	0.9982 (0.0014)
	SP	0.9941 (0.0085)	0.9401 (0.0613)	0.9592 (0.491)	0.9916 (0.0095)

Table S12: Estimated fractions of correct inferences $E_{s,t}$ using the EnsemblCompara GeneTrees data.

**Estimated fraction of correct inferences**					
target species		SC	CE	DM	HS
source species	SC	-	0.166	0.101	0.153
	CE	0.166	-	0.433	0.288
	DM	0.101	0.555	-	0.257
	HS	0.153	0.556	0.341	-

Table S13: Estimated fractions of correct inferences $E_{s,t}$ using the reciprocal-best-hits data.

**Estimated fraction of correct inferences**					
target species		SC	CE	DM	HS
source species	SC	-	0.275	0.214	0.335
	CE	0.274	-	0.800	0.475
	DM	0.214	0.670	-	0.303
	HS	0.335	0.631	0.467	-

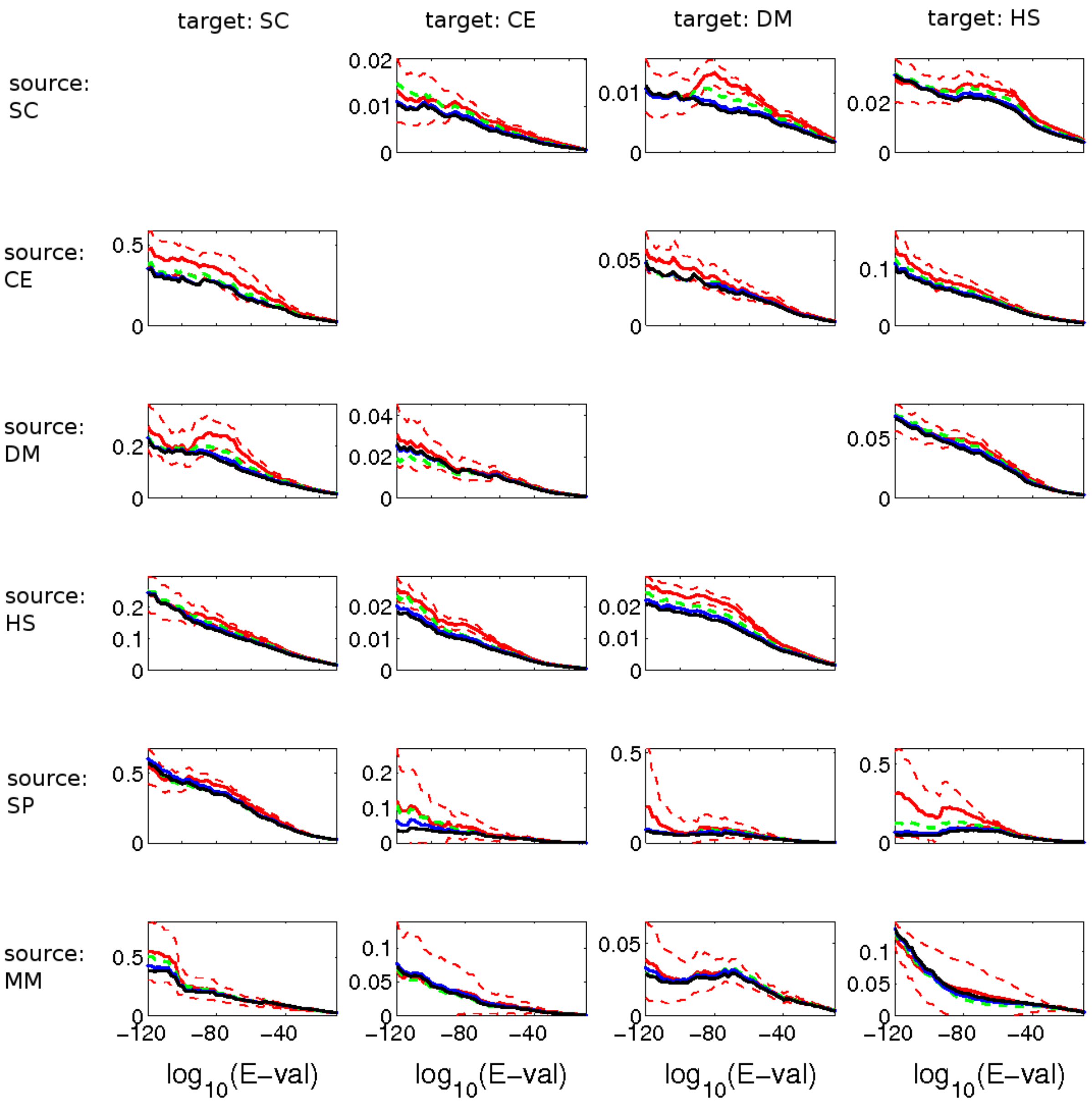

Figure S5: Observed fractions of correct interologs $O_{s,t}$ are largely independent of interaction coverage in the source species. We sub-sample from the source-species interactomes and show mean values of $O_{s,t}$ for the actual data (black curve) and when using only 75% (blue dash-dotted curve), 50% (green dashed curve), and 25% (red curve) of the source-species interactions. We also show the mean ± one standard deviation for the 25% case (dashed red curves). In fact, the values of $O_{s,t}$ actually seem, if anything, to be lower when more interactions are used. Hence, low coverage of the interactions in the source species does not lead to an underestimation of the fraction of correct interologs.

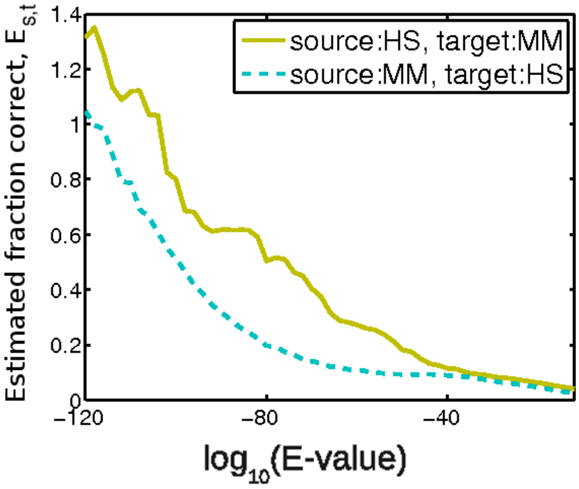

Figure S6: Estimated fraction of correct inferences between *M. musculus* (MM) and *H. sapiens* (HS).

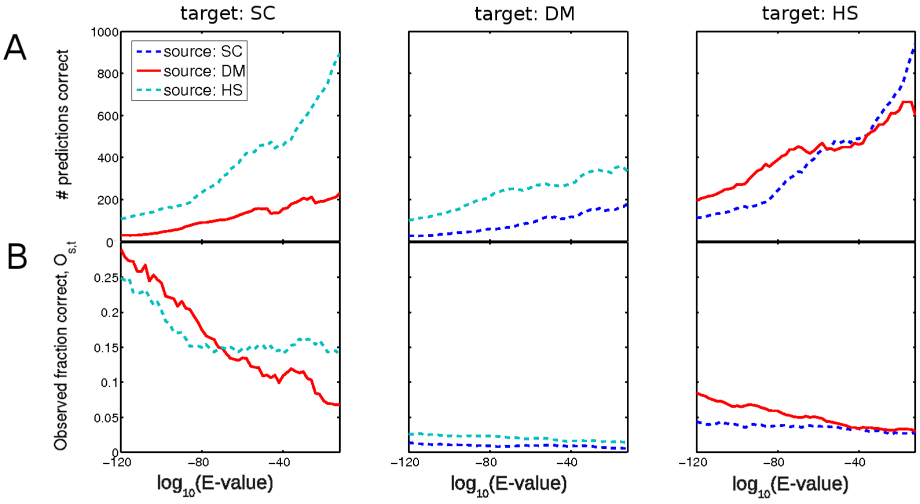

Figure S7: Effects of disallowing inferences from and to large protein families. This figure is the same as for Figure 2 A and B of the main text, except that we only make inferences if each of the four proteins A, B, A', and B' has ten or fewer homologs in the other species. One could argue that the low fraction of correct inferences reported in Figure 2 B of the main text was due in part to allowing inferences from and to large protein families. However, comparing panel B of this figure to Figure 2 B of the main text illustrates that although the fraction deemed to be correct is somewhat higher at lax E-value cut-offs, this comes only at the great expense of a significant decrease in the number of correct predictions (compare panel A of this figure to Figure 2 A of the main text). At more strict E-values, the results are unchanged. In other words, imposing a limit on the sizes of the families has a similar effect to imposing a stricter sequence similarity cut-off.

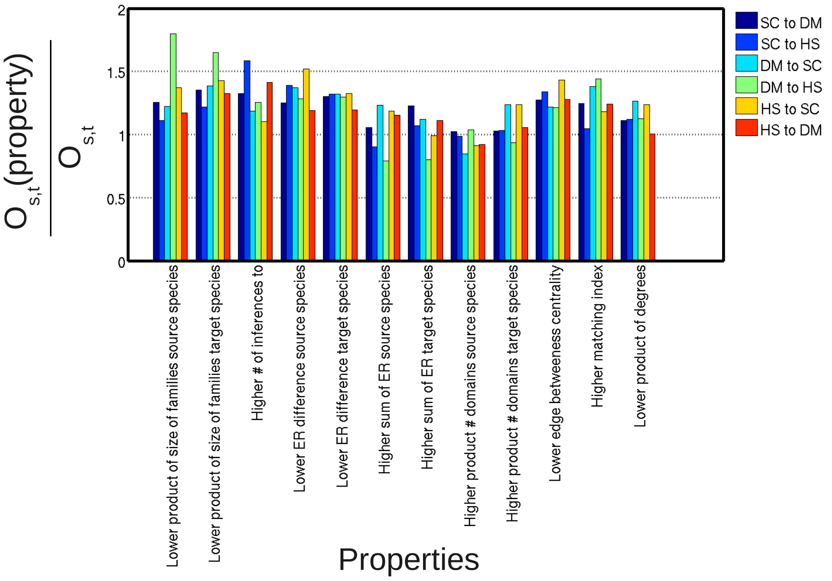

Figure S8: Informativeness of properties for finding conserved interactions. We investigate the helpfulness of certain properties for selecting the correct inferences (see Supplementary text). To give some indication of the utility of the properties for selecting reliable inferences, we calculate $O_{s,t}$ if we select only the half of the inferences with above/below the median of these properties. We denote such quantities by $[O_{s,t}(\text{property})]$ and divide by $O_{s,t}$ for all of our data. Somewhat helpful properties include selecting inferences from and to smaller protein families (as also demonstrated in Figure S7), selecting interologs that are inferred more than once, selecting inferences from or to interacting partners of a similar age (Excess Retention, ER, is a proxy for age), and some properties that assess the local network structure of the interactions in the source species.

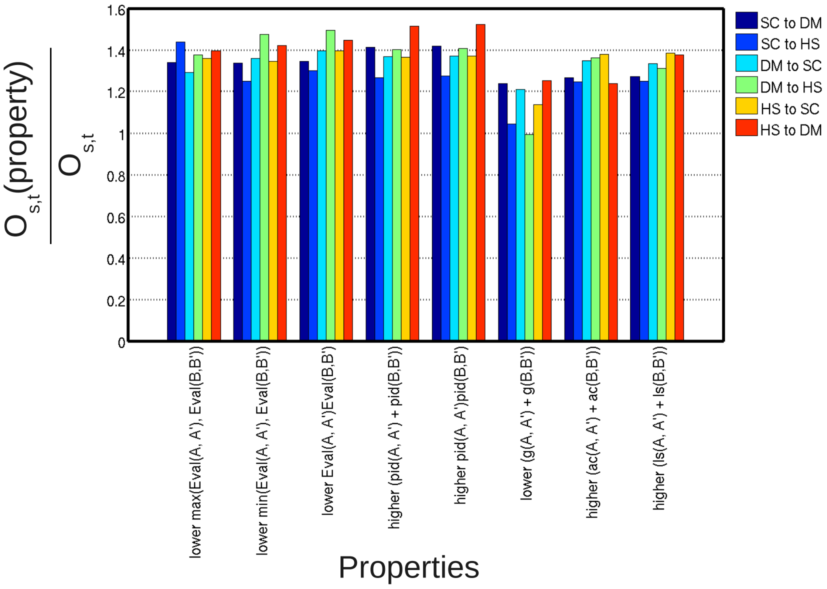

Figure S9: Using the extent of homology to select more reliable inferences: effect of different `blastp` properties. Figures 2–5 of the main text illustrated how the success of interaction inferences varies with the maximum E-value. Here we show, using the list of homology properties in the Supplementary text, how other choices of the extent of homology compare in terms of picking out correct inferences. As for Figure S8 and as explained in the Supplementary text, we select only the half of the inferences with a higher/lower value of these properties and compare the fraction of correct inferences of this subset $[O_{s,t}(\text{property})]$ to the fraction correct $O_{s,t}$ of our whole data set . We find that the properties that aggregate the E-value (E_{val}) or sequence identity values (pid) perform at similar levels of efficacy.

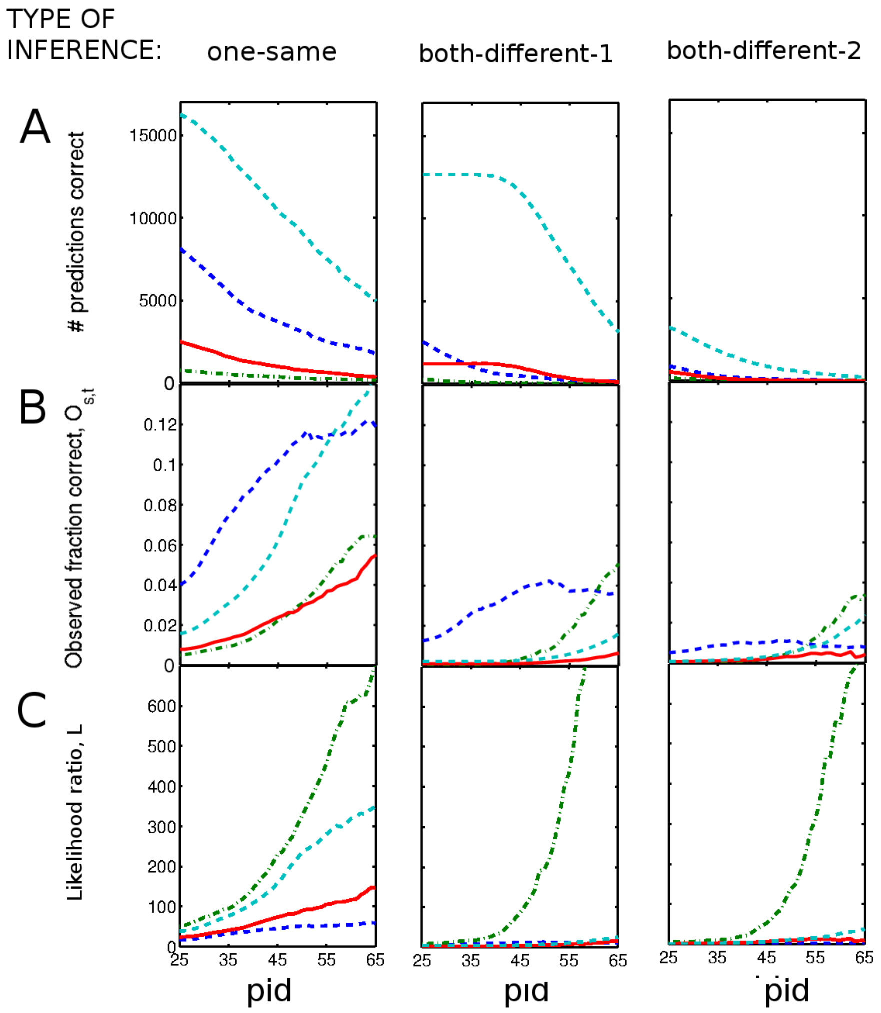

Figure S10: As for Figure 5 of the main text, but for using percentage sequence identity (pid) rather than E-value. For inferences within *S. cerevisiae* (SC), *C. elegans* (CE), *D. melanogaster* (DM), and *H. sapiens* (HS), one-same inferences dominate for (A) the number of correct inferences, (B) the fraction of inferences observed to be correct $O_{s,t}$, and (C) the likelihood L that the inferences are correct. The very large likelihoods for *C. elegans*, particularly for the both-different cases, are due to small-number effects.

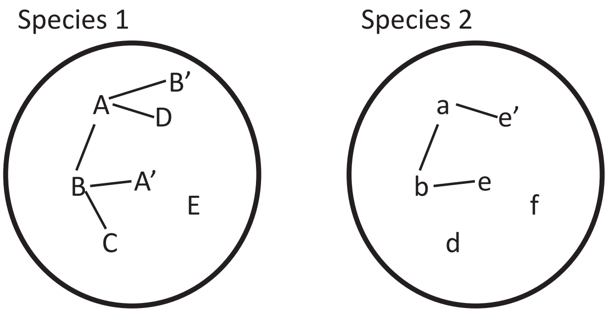

Two species
Species 1 contains proteins A, A', B, B', C, D, and E.
Species 2 contains proteins a, b, d, e, e', and f.

Two proteins are considered homologs if they are the same letter or are the letter'
e.g. E, e, and e' are all homologs.

Interactions are shown as lines (for some proteins, we have no interaction data e.g. E).

Across Species
Infer interactions in Species 2 using Species 1
Infer interaction a – d from A – D
Infer interaction a – b from A – B and A – B' and A' – B
(use only the closest inference A – B)

Inference a – d is incorrect.
Inference a – b is correct.

Accuracy 50%

Difference from Mika and Rost
They would not consider the a – d interaction if the interaction a –d had not been sought explicitly in experiments. Therefore they would achieve 100% accuracy.

Within Species
Infer interactions in Species 1 using Species 1
Infer Interaction B' – C from B – C
A – B from A – B' and A' – B
A' – B from A – B and A – B'
A – B' from A – B and A' – B
A' – B' from A' – B and A – B' and A - B

One-same inferences are underlined.
For both-different-1, make 3 inferences.
For both-different-2, make 0 inferences as the closest inference for all three cases comes from a one-same.

Difference between both-different-1 and Mika and Rost
1) Interactions can only be inferred between different data sets so if the interactions A – B and A – B' and A' – B were from the same data set no inferences would be made.
2) Only consider interactions that have been explicitly tested experimentally. Hence if A' – B' had not been tested for it would not be included.

Figure S11: As for Figure 1 of the main text, including differences between our methodology and that of Mika and Rost.
